# Beyond Melanin: Proteomics Reveals Virulence-Related Proteins in *Paracoccidioides* *brasiliensis* and *Paracoccidioides* *lutzii* Yeast Cells Grown in the Presence of L-Dihydroxyphenylalanine

**DOI:** 10.3390/jof6040328

**Published:** 2020-12-01

**Authors:** Rodrigo Almeida-Paes, Marcos Abreu Almeida, Lilian Cristiane Baeza, Leticia Andrade Mendes Marmello, Monique Ramos de Oliveira Trugilho, Joshua Daniel Nosanchuk, Celia Maria de Almeida Soares, Richard Hemmi Valente, Rosely Maria Zancopé-Oliveira

**Affiliations:** 1 Mycology Laboratory, Evandro Chagas National Institute of Infectious Diseases, Fiocruz, Rio de Janeiro 21040-900, Brazil; marcos.almeida@ini.fiocruz.br (M.A.A.); leticia.marmello@ini.fiocruz.br (L.A.M.M.); rosely.zancope@ini.fiocruz.br (R.M.Z.-O.); 2 Center for Medical and Pharmaceutical Sciences, State University of Western Paraná, Cascavel, Paraná 85819-110, Brazil; lilianbaeza@gmail.com; 3 Molecular Biology Laboratory, Biological Science Institute, Federal University of Goiás, Samambaia Campus II, ICB2, room 206, Goiânia, Goiás 74690-900, Brazil; cmasoares@gmail.com; 4 Laboratory of Toxinology, Oswaldo Cruz Institute, Fiocruz, Rio de Janeiro 21040-900, Brazil; monique.trugilho@cdts.fiocruz.br (M.R.d.O.T.); richardhemmi@gmail.com (R.H.V.); 5 Center for Technological Development in Health (CDTS), Fiocruz, Rio de Janeiro 21040-361, Brazil; 6 Department of Medicine (Division of Infectious Diseases) and Microbiology and Immunology, Albert Einstein College of Medicine, Bronx, NY 10461, USA; josh.nosanchuk@einsteinmed.org

**Keywords:** *Paracoccidioides*, melanin, L-dihydroxyphenylalanine, proteomics, metabolism, virulence

## Abstract

Species of the genus *Paracoccidioides* cause a systemic infection in human patients. Yeast cells of *Paracoccidioides* spp. produce melanin in the presence of L-dihydroxyphenylalanine and during infection, which may impact the pathogen’s survival in the host. To better understand the metabolic changes that occur in melanized *Paracoccidioides* spp. cells, a proteomic approach was performed to compare melanized and non-melanized *Paracoccidioides brasiliensis* and *Paracoccidioides lutzii* yeast cells. Melanization was induced using L-dihydroxyphenylalanine as a precursor, and quantitative proteomics were performed using reversed-phase nano-chromatography coupled to high-resolution mass spectrometry. When comparing melanized versus non-melanized cells, 1006 and 582 differentially abundant/detected proteins were identified for *P. brasiliensis* and *P. lutzii*, respectively. Functional enrichment and comparative analysis revealed 30 important KEGG (Kyoto Encyclopedia of Genes and Genomes) pathways in melanized *P. brasiliensis* and 18 in *P. lutzii*, while differentially abundant proteins from non-melanized cells from these species were involved in 21 and 25 enriched pathways, respectively. Melanized cells presented an abundance of additional virulence-associated proteins, such as phospholipase, proteases, superoxide dis-mutases, heat-shock proteins, adhesins, and proteins related to vesicular transport. The results suggest that L-dihydroxyphenylalanine increases the virulence of *Paracoccidioides* spp. through complex mechanisms involving not only melanin but other virulence factors as well.

## 1. Introduction

Species of the genus *Paracoccidioides* cause a life-threatening systemic disease in humans named para-coccidioidomycosis. *Paracoccidioides brasiliensis* and *Paracoccidioides lutzii* are the primary recognized agents of this infection, although recent taxonomic studies have proposed three additional cryptic species, formerly classified as *P. brasiliensis*: *Paracoccidioides americana*, *Paracoccidioides restrepiensis,* and *Paracoccidioides venezuelensis* [1]. This disease has two major clinical forms, the acute/subacute (or juvenile) and chronic (or adult) forms, and several clinical manifestations, with multi-organ involvement, even in patients without known immunodeficiency [2].

The species *P. brasiliensis* and *P. lutzii* are difficult to distinguish using classical microbiological techniques or by clinical presentation. However, the conidia of *P. brasiliensis* are globular, while *P. lutzii* has elongated conidia [3]. Humans infected with distinct cryptic species have similar clinical presentations [4,5,6], but serologic response to the main antigen used in the diagnosis varies [7]. Regarding virulence, *P. brasiliensis* appears to be more virulent in experimental models of infection, since mice infected with *P. lutzii* develop milder disease and *P. brasiliensis* persists for a prolonged time in the lungs of these animals [8].

In order to cause disease, the species of the genus *Paracoccidioides* have a series of virulence factors that help the fungi invade different tissues and survive the defenses orchestrated by the immune system of the host. The major putative virulence factors of the *Paracoccidioides* genus include the immunodominant gp43, a cell surface and secreted glycoprotein with adhesion and proteinase activities; gp70, a glycoprotein with anti-phagocytic activity; carbohydrates of the cell-wall; and melanin [9].

Melanin is a pigment with stable free radicals produced by diverse organisms, from mammals to bacteria [10]. This biopolymer is usually associated with protection mechanisms against a series of severe conditions and/or toxic compounds present in the environment, such as ultraviolet light, heavy metals and other pollutants, and extremes in temperature [11]. Melanin also protects fungi and other pathogenic microbes during infection [12]. The yeast cells of *Paracoccidioides* spp. produce melanin in vitro under L-dihydroxyphenylalanine (L-DOPA) supplementation and during parasitism [13]. Melanin protects *P. brasiliensis* against phagocytosis and antifungal drugs [14]. However, there are no data on the mechanism(s) of action associated with these protective phenotypes in melanized *Paracoccidioides* yeast cells.

Proteomic approaches have been applied to the genus *Paracoccidioides* to elucidate several aspects related to the adaptation and survival of the fungus in the host, such as macrophage interaction [15], response to hypoxia [16], and metabolism under carbon starvation [17]. Therefore, we compared the proteome of melanized and non-melanized (that is, cultured in the presence or absence of L-DOPA, respectively) *P. brasiliensis* and *P. lutzii* strains to identify possible molecular mechanisms that explain the higher survival of melanized cells under harsh conditions.

## 2. Materials and Methods

### 2.1. Fungal Strains and Culture Maintenance

The reference strains *P. brasiliensis Pb*18 [18] and *P. lutzii Pb*01 (ATCC- MYA-826) [19] were used in the experiments. Yeast cells were sub-cultured in FavaNetto Agar medium (0.3% (*w*/*v*) proteose peptone (Difco Laboratories, Sparks, MD, USA); 1% (*w*/*v*) peptone (Difco Laboratories, Sparks, MD, USA); 0.5% (*w*/*v*) beef extract (Difco Laboratories, Sparks, MD, USA); 0.5% (*w*/*v*) sodium chloride (Sigma-Aldrich, Co., St. Louis, MO, USA); 0.5% (*w*/*v*) yeast extract (Difco Laboratories, Sparks, MD, USA), 4% (*w*/*v*) glucose (Sigma-Aldrich, Co., St. Louis, MO, USA), 1.8% (*w*/*v*) agar (Difco Laboratories, Sparks, MD, USA)) and incubated at 37 °C on a weekly basis.

### 2.2. Melanin Production

*Paracoccidioides* yeast cells were harvested from seven-day-old solid FavaNetto cultures and washed three times with phosphate-buffered saline (PBS). Melanized cultures of the strains were obtained as follows: firstly, the harvested and washed cells were grown in FavaNetto liquid medium (same composition as above, but without agar) at an initial density of 1 × 10^6^ cells/mL for six days at 37 °C, under agitation (150 rpm). After this first incubation, 1 × 10^8^ cells were transferred to a chemically defined minimal medium (15 mM glucose, 10 mM MgSO_4_, 29.4 mM K_2_HPO_4_, 13 mM glycine, and 3.0 mM thiamine (all from Sigma-Aldrich Co., San Luis, MO, USA), pH 5.5] supplemented with 1 mM L-dihydroxyphenylalanine (L-DOPA) (Sigma-Aldrich, Co., San Luis, MO, USA). Control non-melanized cultures were performed in the same conditions, but without L-DOPA supplementation into the minimal medium. All cultures were incubated in a rotary shaker (Nova Ética, Vargem Grande Paulista, SP, Brazil) for six days at 150 rpm and 37 °C, collected by centrifugation (10,000× *g*), and washed three times in PBS. Five biological replicates were prepared for each condition.

### 2.3. Protein Extraction

Extracts containing total protein contents from melanized and non-melanized *Pb*18 and *Pb*01 strains were obtained by mechanical cellular disruption under liquid nitrogen. Extraction buffer (20 mM Tris-HCl pH 8.8, 2 mM CaCl_2_), supplemented with protease inhibitors (Complete Mini^®^, Roche Diagnostics, Manheim, Germany) and zirconia beads of 0.5 mm diameter (BiospecProducts, Bartlesville, OK, USA), were added to the fungal lysates. A vigorous agitation of this mixture was performed on a Mini-Beadbeater (Biospec products) for five cycles of 30 s, separated by 1 min ice bath incubations. After this procedure, the material was centrifuged (10,000× *g*) for 15 min at 4 °C and the Bradford method was employed to determine protein content, using bovine serum albumin (Sigma-Aldrich, Co.) for the generation of standard curves [20]. The protein profiles of the fungal extracts were evaluated by sodium dodecyl sulfate-polyacrylamide gel electrophoresis (SDS-PAGE), as described [21].

### 2.4. Sample Processing for Shotgun Proteomics

For each sample, the volume equivalent to 100 μg was dried on a centrifugal vacuum concentrator (SpeedVac, Thermo Fischer Scientific, Waltham, MA, USA) and then submitted to trypsin digestion. Samples were initially suspended in 20 μL of 0.4 M ammonium bicarbonate, 8 M urea, followed by the addition of 5 μL of 0.1 M dithiothreitol and incubation at 37 °C for 30 min. Then, 5 μL of 0.4 M iodoacetamide were added and incubated for 15 min at room temperature in the dark. Samples were diluted by the addition of 130 μL of Milli-Q water followed by trypsin (Promega, Madison, WI, USA) addition at 1/50 (m/m) of enzyme to substrate and sequential incubation for 16 h at 37 °C and 45 min at 56 °C; the reaction was stopped with 20 μL of 10% (*v*/*v*) formic acid. Samples were desalted with in-lab generated columns packed with Poros R2 resin (Life Technologies). Columns were initially activated with 100% acetonitrile (CH_3_CN), followed by equilibration with 1% (*v*/*v*) trifluoroacetic acid (TFA). Samples were applied to the columns and subjected to five washes with 0.1% TFA solution. The elution was carried out with four washes of 0.1% TFA in 70% CH_3_CN. Samples were dried on a centrifugal vacuum concentrator (SpeedVac) and stored at −20 °C until use. Prior to mass spectrometry (MS), each sample was suspended in 20 μL of 1% formic acid, and its peptide concentration was estimated by absorbance measurement at 280 nm (1.000 absorbance unit ≅ 1.000 μg/μL) on a NanoDrop 2000 spectrophotometer (Thermo Fischer Scientific); all samples were normalized to 0.675 μg/μL.

### 2.5. Mass Spectrometry Analysis

The desalted tryptic digests from the five biological replicates from the strains *P. brasiliensis Pb*18 and *P. lutzii Pb*01 were each analyzed in three technical replicates by reversed-phase nano-chromatography coupled to high-resolution nano-electrospray ionization mass spectrometry. Chromatography was performed using a Dionex Ultimate 3000 RSLCnano system (Thermo Fischer Scientific). Samples (4 µL per run) were initially applied, at 2 μL/min of 0.1% (*v*/*v*) formic acid in water, to a 2-cm-long trap column (100 μm inner diameter), packed with Magic C18-AQ 200 Å 5 μm matrix (MichromBioresources, Auburn, CA, USA). Next, peptides were submitted to chromatographic separation on a 26-cm-long column (75 μm inner diameter), packed with ReproSil-Pur C18-AQ 120 Å 1.9 μm matrix (Dr. Maisch GmbH, Germany) directly into a laser-pulled capillary with a 4 μm tip. Fractionation was performed at 200 nL/min having 0.1% (*v*/*v*) formic acid in water and 0.1% (*v*/*v*) formic acid in acetonitrile as mobile phases A and B, respectively. Elution was carried out on a gradient from 2% to 40% B for 162 min; concentration was increased to 80% B in 4 min and maintained for 2 min more. The eluted peptides were introduced directly into a Q Exactive Plus Orbitrap instrument. Ionization was achieved by applying 1.9 kV to the source, setting the capillary temperature to 250 °C, and alternate current (radiofrequency) level of the S-lenses at 60 V. The complete MS1 scans (300 to 1500 *m/z*) were acquired in the profile mode with one micro-scan at 70,000 resolution and an automatic gain control (AGC) target value of 1 × 10^6^ with a maximum injection time of 100 ms. The 12 most intense precursor ions within both isolation window and offset of 2.0 and 0.5 *m/z*, respectively, were selected for HCD (higher energy collision dissociation) fragmentation with a collision energy normalized to 30%. The MS2 spectra (200 to 2000 *m/z*) were acquired in centroid mode with one micro-scan at 17,500 resolution and an AGC target value of 5×10^4^ with a maximum injection time of 50 ms. Dynamic exclusion was set to 60 s, whereas peaks with unassigned charge or those with z = 1 were rejected.

### 2.6. Protein Identification and Quantitation Following Mass Spectrometry

Data were analyzed using the PatternLab for Proteomics 4.0 pipeline [22]. Separate reference databases were downloaded for each species from the UniProt proteomes repository, comprising 8811 entries for *Pb*01 (https://www.uniprot.org/proteomes/UP000002059) and 8399 entries for *Pb*18 (https://www.uniprot.org/proteomes/UP000001628). The “Generate Search Database” tool was used to add to each database common contaminants and decoy (reverse) sequences for all entries. MS data from each species sample runs were searched against their specific database using the Comet search tool [23], which is integrated into PatternLab. The parameters were set as follows: precursor mass error tolerance of 40 ppm, trypsin as the enzyme, semi-tryptic cleavage, maximum number of cleavage loss of 2, cysteine carbamido-methylation (+57.02146 Da) as fixed modification, and deamination of asparagine and/or glutamine (+0.98400 Da) as variable modifications. Values for fragment bin tolerance, fragment bin offset, and theoretical fragment ions were 0.02, 0, and default peak shape (flanking peaks), respectively. The mass spectrometry proteomics data have been deposited to the ProteomeXchange Consortium (http://proteomecentral.proteomexchange.org) via the PRIDE partner repository [24] and received the dataset identifier PXD021711. Spectral validation was performed using SEPro algorithm (Search Engine Processor) [25]; false discovery rate (FDR) was calculated from the number of decoy sequences identified, so that a maximum limit of 1% FDR, at peptide and protein levels, was established and only identifications with a mass error tolerance of 5 ppm were accepted. Unique peptide spectral count analysis was used for the quantitative comparisons between melanized and non-melanized cells of each species in the TFold module of PatternLab for Proteomics; normalization was done by total spectral count. Only proteins that were detected in at least three (out of five) biological replicates, for each condition, were submitted to relative quantitation with cut-off values for Benjamini–Hochberg q-value, F-stringency, and L-stringency of 0.01, 0.03, and 0.60, respectively; proteins that satisfied both the fold-change and statistical (*p*-value) criteria were further submitted to protein interaction and metabolic pathway analyses.

### 2.7. Bioinformatic Analyses

The Uniprot resource for protein sequence and annotation (https://www.uniprot.org/) was used to describe functions of proteins with quantitative differences on both conditions of each *Paracoccidioides* species. Enrichment analyses of Kyoto Encyclopedia of Genes and Genomes (KEGG) pathways (https://www.genome.jp/kegg/pathway.html) were used to functionally categorize the regulated proteins and interactions between proteins were studied on the STRING v.11 server [26], available at https://string-db.org/. Proteins associated with virulence, stress response, and pathogenesis of paracoccidioidomycosis were retrieved from indexed publications [9,27,28,29,30,31,32,33] and searched into the proteomic data generated in this study. Heat maps were constructed using the GraphPad Prism 8.4.2 software.

## 3. Results

### 3.1. Fungal Melanization and Protein Extract Control

**Figure 1:**
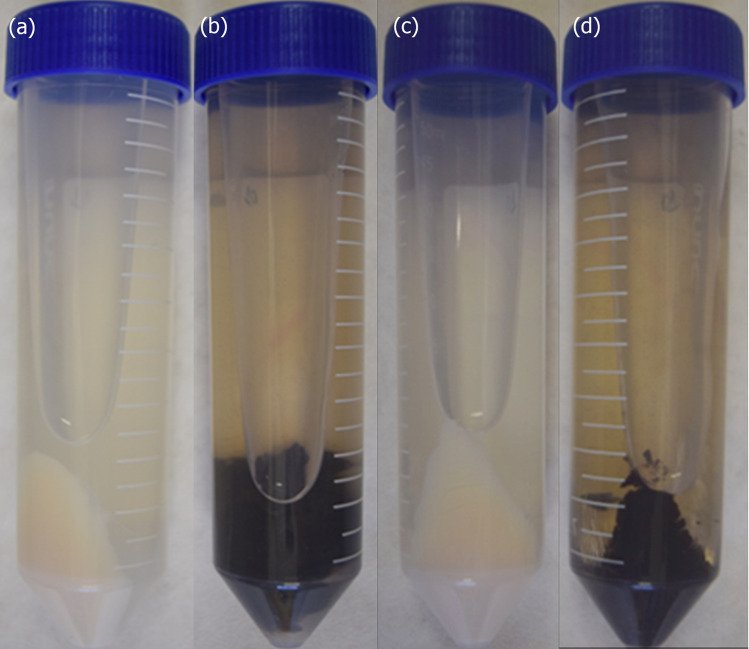
L-dihydroxyphenylalanine (L-DOPA) induces melanization in *Paracoccidioides* species. Pellets of *Paracoccidioides brasiliensis Pb*18 yeast cells after six days of growth in minimal medium alone (**a**) or supplemented with L-DOPA (**b**). Pellets of *Paracoccidioides lutzii Pb*01 yeast cells after six days of growth in minimal medium (**c**) or supplemented with L-DOPA (**d**).

Both *Pb*18 and *Pb*01 strains, *P. brasiliensis* and *P. lutzii*, respectively, produced melanized cells after six days of incubation at 37 °C, exclusively in the presence of L-DOPA (Figure 1). The quality of the protein extracts obtained from melanized and non-melanized cells was evaluated by SDS-PAGE under reducing conditions (Figure S1). Proteins within a broad range of molecular mass were observed on both species; for some bands, a variation of the staining intensity was observed, indicating a possible differential abundance of proteins.

### 3.2. Proteomic Analysis of Melanized and Non-Melanized Cells

The proteomic approach identified 2586 protein entries for the *P. brasiliensis Pb*18 strain and 1962 for the *P. lutzii Pb*01 strain. In the *Pb*18 proteome, 471 proteins were exclusively detected in non-melanized cells and 72 in melanized cells. Similarly, 275 proteins were only detected in the proteome of non-melanized *Pb*01 cells and 71 in melanized cells proteome (Figure S2). Among the 2043 proteins detected in extracts from both melanized and non-melanized Pb18 cells, 463 presented a statistically-supported (*p*- and *q*-values < 0.01) differential abundance between the two conditions tested; 245 were more abundant in non-melanized and 218 in melanized cells (Table S1-“Conditions&Volcano Plot” and “Blue” sheets). Regarding *P. lutzii*, 1,=616 proteins were detected in proteomes from the two studied conditions; 236 of these presented a statistically-supported (*p*- and *q*-values < 0.01) difference in abundance: 108 were more abundant in melanized cells and 128 in non-melanized cells (Table S2—“Conditions&Volcano Plot” and “Blue” sheets).

### 3.3. Enriched Pathways

The proteins differentially abundant and exclusively detected, for both culture conditions of each *Paracoccidioides* species, were clustered and analyzed using the STRING database. Both *Pb*18 and *Pb*01 proteins yielded networks (Figure S3) with more interactions than expected, presenting protein–protein interaction (PPI) enrichment values of less than 10^−16^ and 2.77 × 10^−9^, respectively. Pathways related to carbon, nucleotide, amino acid metabolisms, and other biological processes were enriched in the species studied, as presented in Table 1.

#### 3.3.1. Carbon Metabolism

**Figure 2:**
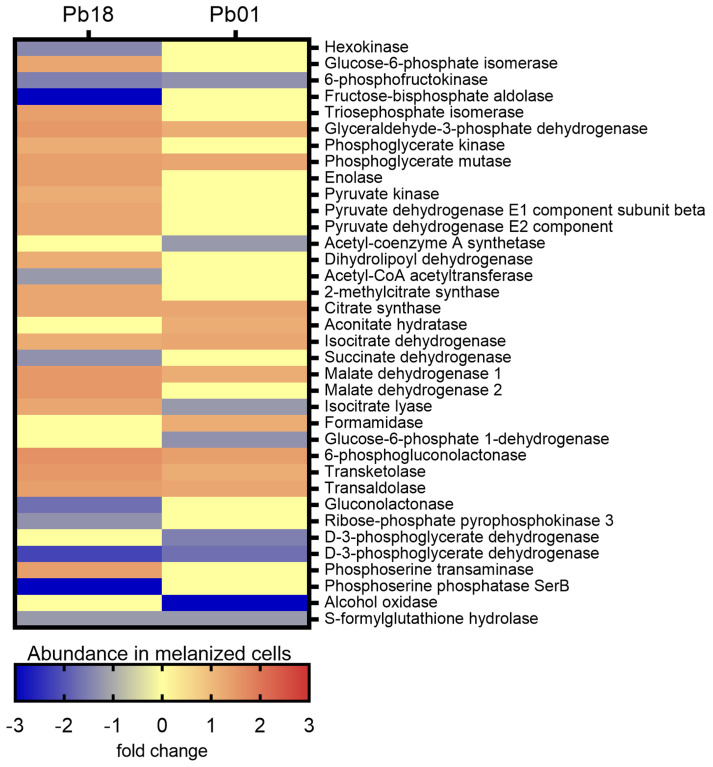
Comparative heatmap of the abundance in melanized cells of proteins from carbon metabolism pathways enriched in *P. brasiliensis Pb*18 and *P. lutzii Pb*01.

The KEGG pathways enrichment analysis revealed 49 proteins associated with carbon metabolism in *Pb*18 and 24 in *Pb*01 (Figure S4). Twelve proteins were common to the two species. Among the *Pb*18 proteins, 37 were more abundant in melanized cells, nine were more abundant in non-melanized cells, and three were exclusively detected in non-melanized cells. All proteins associated with the oxidative phosphorylation were more abundant in melanized cells, as well as the late enzymes of the glycolytic pathway, isocitrate lyase, one of the key enzymes of the glyoxylate cycle, and the enzymes transketolase and trans-aldolase of the non-oxidative phase of the pentose phosphate pathway (Figure 2).

Most enzymes related to methane metabolism and the citrate cycle were more abundant in non-melanized cells. In *Pb*01, transketolase and trans-aldolase were also enriched, as well as some enzymes from glycolysis and citrate cycle. However, the key enzyme of glycolysis, hexokinase (PADG_03813), was less abundant in melanized cells of *Pb*18 strain, as well as phosphofructokinase (PADG_00192 and PAAG_01583), in melanized cells of both strains studied (Figure 2). Although the oxidative phosphorylation was not enriched in *Pb*01, the beta and delta subunits of ATP synthase (PAAG_08037 and PAAG_05605) were more abundant in melanized cells (fold changes of 1.3 and 1.7, respectively), similarly to *P. brasiliensis*. In contrast, isocitrate lyase was less abundant in melanized cells of *P. lutzii*. The enzymes involved in β-oxidation of fatty acids enoyl-CoA hydratase and acyl-CoA dehydrogenase were found to be more abundant in melanized *Pb*18. However, this pathway was not enriched in the *Pb*01 proteome. In fact, the only enzyme related to fatty acid metabolism with differential abundance in this strain was 3-ketoacyl-CoA thiolase (PAAG_02664), with a fold change of −1.3.

#### 3.3.2. Nucleotide Metabolism

**Figure 3:**
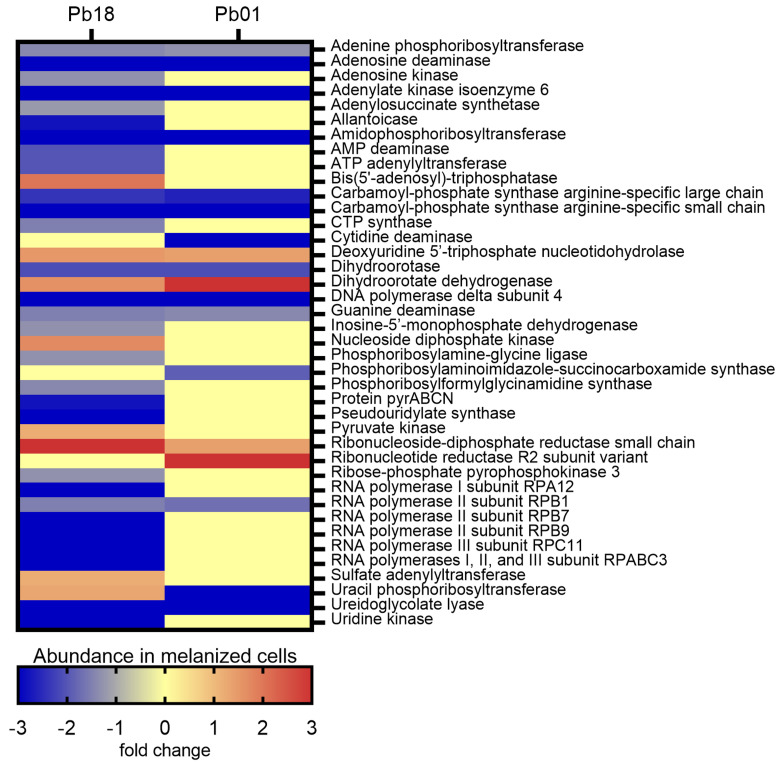
Comparative heatmap of the abundance in melanized cells of proteins from nucleotide metabolism pathways enriched in *P. brasiliensis Pb*18 and *P. lutzii Pb*01.

The KEGG pathways enrichment analysis showed 37 proteins associated with nucleotide metabolism in *Pb*18 and 18 in *Pb*01 (Figure S5). Fifteen proteins were common to the two strains studied. In general, most enzymes from both purine and pyrimidine synthesis were more abundant (14 proteins in the *Pb*18 proteome and six in the *Pb*01 proteome) or exclusively detected (15 proteins in the *Pb*18 proteome and eight in the *Pb*01 proteome) in non-melanized cells of both species. The abundance profile of differentially abundant proteins related to the nucleotide metabolism was similar for the two *Paracoccidioides* strains analyzed, except for uracil phosphoribosyl-transferase (PADG_01100 and PAAG_06643), which was found to be more abundant in *Pb*18 melanized cells but less abundant in melanized cells of *Pb*01 (Figure 3).

#### 3.3.3. Amino Acid Metabolism

**Figure 4:**
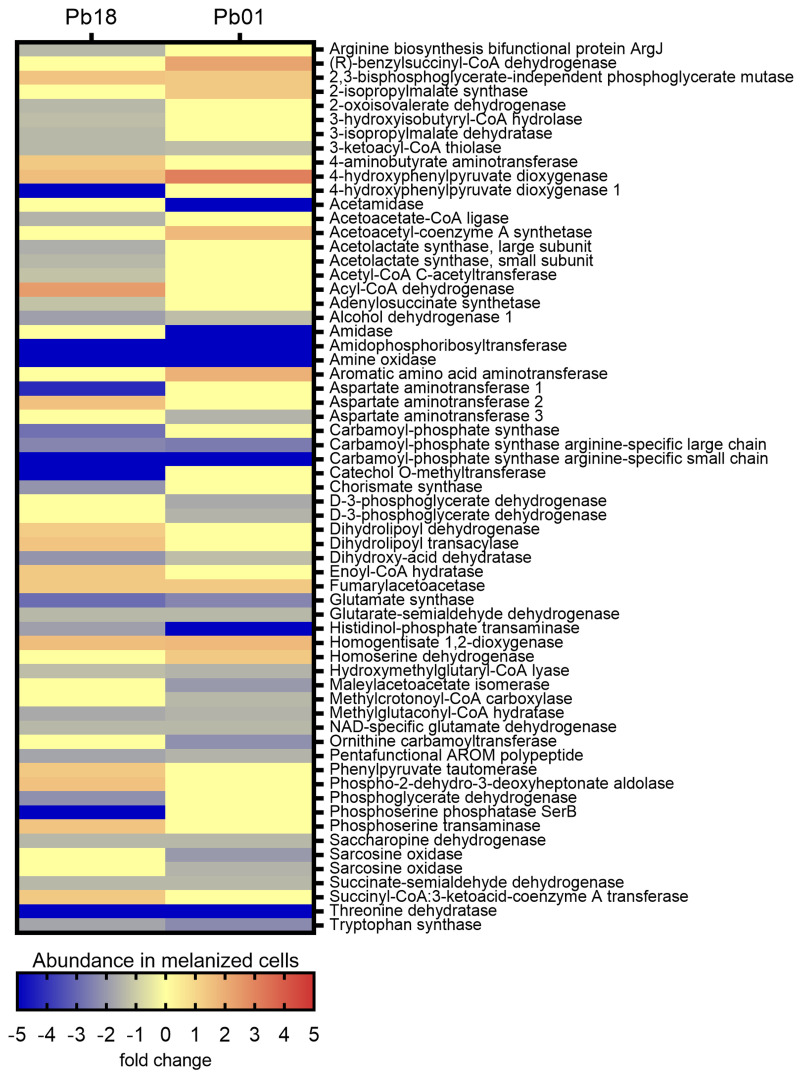
Comparative heatmap of the abundance in melanized cells of proteins from amino acid metabolism pathways enriched in *P. brasiliensis Pb*18 and *P. lutzii Pb*01.

The KEGG pathways enrichment analysis showed 65 exclusive proteins or proteins with differential abundance associated with amino acid metabolism in *Pb*18 and 51 in *Pb*01 (Figure S6). Twenty-two proteins were common to the two organisms. In *Pb*18, most pathways related to amino acid metabolism were more abundant in non-melanized cells, except for six enzymes of the tyrosine metabolism, another six from the valine, leucine, and isoleucine degradation, as well as two enzymes from the glycine, serine, and threonine metabolism and one from the phenylalanine, tyrosine, and tryptophan biosynthesis. A similar profile was observed in the *Pb*01 proteomes, with a predominance of enzymes more abundant in non-melanized cells. Melanized cells presented five abundant proteins related to tyrosine metabolism, two related to valine, leucine, and isoleucine degradation, two enzymes related to glycine, serine, and threonine metabolism, and one related to valine, leucine, and isoleucine biosynthesis. Supporting that degradation of amino acids generates precursors for the citrate cycle, we observed that the enzyme 3-oxoacid CoA transferase (PADG_04939), related to the degradation of leucine to acetyl CoA, was more abundant in the melanized *Pb*18 proteome. Enzymes related to the tyrosine degradation to fumarate presented differential abundance among melanized and non-melanized cells of both strains. They include fumarylaceto-acetase (PADG_08465 and PAAG_ 08163), homogentisate 1,2-dioxygenase (PADG_08466 and PAAG_08164), 4-hydroxyphenylpyruvate dioxygenase (PADG_08468 and PAAG_08166), phenylpyruvate tautomerase (PADG_03671), and two isoforms of aspartate aminotransferase (PADG_03686 and PADG_01404). Surprisingly, a third aspartate aminotransferase isoform (PAAG_02603) was less abundant in melanized *Pb*01 yeast cells (Figure 4).

#### 3.3.4. Other Metabolic Pathways

The enrichment analysis revealed another ten KEGG pathways in the *Pb*18 proteome: spliceosome, RNA degradation, RNA transport, protein processing in the endoplasmic reticulum, phagosome, aminoacyl-t-RNA biosynthesis, proteasome, basal transcription factors, soluble N-ethylmaleimide-sensitive-fusion attachment protein receptor (SNARE) interactions in vesicular transport, and ubiquitin-mediated proteolysis (Figure S7a). All proteins with differential abundance associated with SNARE interactions in vesicular transport and the majority (72.7%) of proteasome proteins were more abundant in melanized cells. Half the proteins associated with protein processing in the endoplasmic reticulum were also more abundant in melanized cells. The majority of proteins from the other seven pathways were more abundant in non-melanized cells. RNA degradation and RNA transport were also enriched in proteomes of *Pb*01, but not the other eight pathways observed in *Pb*18. In fact, four pathways were enriched in *Pb*01: RNA degradation, RNA transport, mRNA surveillance pathway, and peroxisome. Except for RNA degradation, most proteins from the other pathways were less abundant in melanized yeast cells (Figure S7b). Heat maps of each KEGG pathway enriched on both *Pb*18 and *Pb*01 strains are presented in Figure S8.

### 3.4. Virulence-Associated Proteins

**Figure 5:**
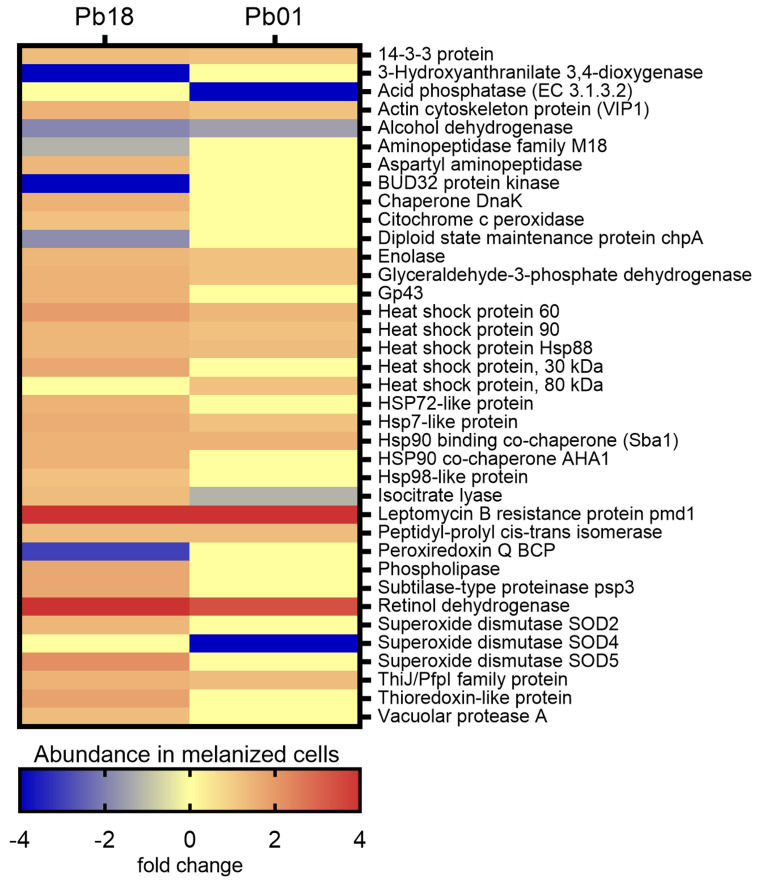
Comparative heatmap of the abundance in melanized cells of virulence-related proteins in *P. brasiliensis Pb*18 and *P. lutzii Pb*01.

A search was conducted for the proteins exclusively detected or with differential abundance in melanized and non-melanized cells of *Pb*18 and *Pb*01 strains. Figure 5 depicts the abundance of virulence-related proteins in melanized cells of *Pb*18 and *Pb*01 strains. Among 1006 proteins of *Pb*18, 34 were previously associated with fungal virulence, stress response, and/or pathogenesis of paracoccidioido-mycosis. As for *Pb*01, 18 proteins fulfilling the same criteria aforementioned were observed among 582 proteins.

As depicted in Figure 5, most of the 37 virulence-related proteins observed in both *Paracoccidioides* species were more abundant in their melanized cells. Noteworthy, eight heat shock proteins and two co-chaperones, four proteolytic enzymes, a protein involved in multidrug resistance (Leptomycin B resistance protein pmd1), and some adhesins such as 14-3-3 protein, glyceraldehyde-3-phosphate dehydrogenase, enolase, and actin cytoskeleton protein VIP1 stand out among the abundant virulence-related proteins detected in melanized cells. The immunodominant gp43 antigen was also more abundant in melanized cells of the Pb18 yeast cells. The single protein related to virulence that was less abundant in melanized cells of both strains was alcohol dehydrogenase, which has adhesin properties.

## 4. Discussion

Members of the genus *Paracoccidioides* change their protein expression, and consequently their metabolism and virulence, as a response to physiological conditions induced in experimental settings, such as copper overload [34], copper deprivation [35], acetate availability [31], osmotic stress [36], among others. The present work demonstrates that *P. brasiliensis* and *P. lutzii*, when grown in vitro in the presence of L-DOPA, a substance potentially available for the fungus during parasitism, not only produce melanin, as already described [13,14], but also undergo metabolic shifts and present differential abundance of proteins previously related with fungal virulence.

The most studied human pathogenic fungus regarding melanization is *Cryptococcus neoformans*. In this species, L-DOPA only changes the expression of eight genes, most of them related to stress response [37]. On the other hand, *P. brasiliensis* and *P. lutzii* have significant changes on overall metabolism, and protein expression when L-DOPA is available during fungal growth. The differences between these two fungi might be related to the methodologies used in the studies: while in *C. neoformans* the early response (4 h incubation) to L-DOPA was evaluated at the transcript level, here the proteome of fully melanized cells (six days of incubation with L-DOPA) was evaluated.

The concentration of L-DOPA used in this study (1 mM) is widely used for several groups that study in vitro fungal melanization [37,38,39,40,41]. The amount of L-DOPA in infected tissues is lower than that used in experimental conditions [42], but it is continuously renewed in tissues whereas it is depleted over time in culture as it is incorporated by the fungus. However, for in vitro proteomics, a large number of cells is necessary to yield protein amounts suitable for analysis, a number also higher than that usually found in infected tissues of patients with paracoccidioido-mycosis. Therefore, the use of physiological amounts of L-DOPA in our experimental conditions using a high number of cells would be challenging, as previously demonstrated with *C. neoformans* [37]. Further studies are required to address whether these results correlate with in vivo conditions.

In the proteomic approach herein performed, *P. brasiliensis* displayed more changes than *P. lutzii* in response to L-DOPA. In fact, *P. brasiliensis* presented almost two-fold more proteins with differential abundance than *P. lutzii* and, besides carbon, amino acid, and nucleotide metabolisms, showed eight enriched pathways not observed in *P. lutzii*, which had only two exclusive enriched pathways. These differences, however, could not be associated with the known phenotypic differences between *P. brasiliensis* and *P. lutzii*. Among the enriched processes observed only in *P. brasiliensis*, proteasome induction also occurred in the *Pb*18 strain during keratinocyte infection [43], with the alpha subunit of the proteasome (PADG_02735) detected in both experimental approaches. In addition, *P. brasiliensis* melanized yeast cells had a higher abundance of proteins associated with vesicular transport. As previously described [44], this species is known to produce extracellular vesicles harboring proteins that could modulate the immune system, proteins associated with stress response, and enzymes involved in the acquisition of nutrients during parasitism, like lipases and the subtilase-type proteinase psp3 (PADG_07422), also more abundant in the total proteome of *Pb*18 melanized yeast cells described in this study. In *Pb*01, most peroxisomal proteins were less abundant in melanized cells. Peroxisomes are associated, among other functions, with the reduction of reactive oxygen species [45], a role that might be addressed by melanin of *P. lutzii* cells grown in the presence of L-DOPA.

To the best of our knowledge, this is the first comparative proteomic approach in melanized and non-melanized cells of two phylogenetically related fungal species. We have previously compared proteomes of non-melanized phylogenetically related fungal species. For example, in pathogenic species of the genus *Sporothrix*, enzymes related to amino acid metabolism and cell-wall remodeling are more abundant in the highly virulent *Sporothrix brasiliensis* species, whereas the glycolytic pathway is more active in *Sporothrix schenckii* and *Sporothrix globosa*. Moreover, the number of proteins with differential abundance is lower than that observed in this study [46]. We previously compared the proteomic profile of four distinct phylogenetic species of *Paracoccidioides*, including the *Pb*01 strain used in this study, where 301 proteins presented differential abundance [47]. The lower number of proteins may be explained by the method used in the previous study, two-dimensional gel electrophoresis and in-gel digestion, which is less sensitive than the method used in this study.

Proteins related to carbon metabolism were more abundant in melanized cells of both strains studied. The citrate cycle was enriched in both strains; however, oxidative phosphorylation was only enriched in *Pb*18. A similar behavior was observed when different *Paracoccidioides* species were grown in the presence of acetate, a common carbon source within macrophages [31]. The high abundance of proteins related to the electron transport chain and oxidative phosphorylation of *P. brasiliensis* related to *P. lutzii* has been previously demonstrated [47], which could explain the results from the present study. The glyoxylate cycle appears to be differentially regulated by L-DOPA in the two studied strains since isocitrate lyase is more abundant in melanized *Pb*18 and less abundant in melanized *Pb*01. The glyoxylate cycle is associated with virulence in some human pathogenic fungi [48,49], and isocitrate lyase appears to have a role in adhesion and colonization of host tissues by *P. brasiliensis* [50], reinforcing the role of L-DOPA in the virulence enhancement of this species. The enzymes enoyl-CoA hydratase (PADG_01209) and acyl-CoA dehydrogenase (PADG_02244) were more abundant in melanized *Pb*18, indicating β-oxidation of fatty acids in this strain, which also occurs when acetate is available for fungal growth [31], and during copper deprivation [35] or experimental infection [51]. It was noteworthy that the enzymes related to ethanol production did not present differential abundance between melanized and non-melanized cells of both strains tested. Ethanol production is regulated in *Paracoccidioides* spp. by several factors, including growth with acetate as a carbon source [31], carbon starvation [17], response to antifungal molecules [28], and during macrophage or murine infection [51,52].

The profile of enzymes related to amino acid metabolism was also similar between *Pb*18 and *Pb*01 strains. Degradation of amino acids provides precursors for the citrate cycle (Figure S9). Amino acids are a potential nutrient source for fungal growth during parasitism. Our proteomic data show that both melanized strains have the enzymatic machinery to yield fumarate from tyrosine. Another fate of tyrosine is its conversion to L-DOPA by the enzyme tyrosinase. This enzyme was not detected in our proteomic approach, despite its theoretical role in melanin production [14]. One hypothesis to explain this result is that tyrosinase may be regulated in the early stages of fungal melanization and, therefore, cannot be detected after the fungus is fully melanized. In addition, we cannot exclude the possibility of the participation of proteins without known functions in melanin production by *Paracoccidioides* spp. In fact, approximately 60% of the total proteins predicted in the genomes of *P. brasiliensis Pb*18 and *P. lutzii Pb*01 do not have annotated functions. In addition, even after a functional classification of *P. lutzii* hypothetical proteins with gene ontology terms and protein domain annotations, more than 2500 proteins remained without a predicted function [53].

L-DOPA is described as suppressing *Madurella mycetomatis* growth, even at 0.1 mM [40], and to inhibit the growth of *C. neoformans* in concentrations above 1 mM [37]. This last concentration was not able to inhibit the growth of the two *Paracoccidioides* strains herein used, but some proteomic data described in this study indicate that melanized yeast cells of *P. brasiliensis Pb*18 have a lower transcriptional capacity than non-melanized cells. These data include the absence of several basal transcription factors in melanized cells, the lower abundance of enzymes related to the nucleotide metabolism, including five subunits of three different RNA polymerases, and the lower abundance of proteins associated with spliceosome. Interestingly, this behavior was not observed with the *P. lutzii Pb*01 strain, which only presented one less abundant subunit of the RNA polymerase (PAAG_05397). These results indicate that the two *Paracoccidioides* strains used in this study have distinct responses to L-DOPA availability. Whether these differences are strain-dependent or reflect the species-specific biological behavior of *P. brasiliensis* and *P. lutzii* remains to be elucidated.

Melanized fungal cells are more resistant to several harsh conditions faced during environmental or parasitic growth. Much of this protective effect can be explained by the biophysical properties of melanin, such as absorbance of ultraviolet light, covalent and non-covalent interaction with molecules, and scavenging of free radicals [11]. The results of the proteomic approach used in this study revealed that, besides the well-established function of melanization promotion [13,14], L-DOPA also induces the expression of several other molecules related to fungal virulence in *Paracoccidioides* spp. Similar behavior was observed for the *Pb*18 strain treated with low doses of nitric oxide [54]. We found that *Pb*18 presented more virulence-related proteins, with higher abundance than *Pb*01, which can be related to the higher virulence of this strain in experimental mice models of paracoccidioido-mycosis [8].

Eight heat shock proteins and two co-chaperones were more abundant in melanized cells. Heat shock proteins can be induced in response to biotic and abiotic factors, including high temperatures [55]. Both cultures, supplemented or not with L-DOPA, were grown in the same incubator, at the same time, discarding the hypothesis that changes in temperature could have influenced our results. Since heat shock proteins are immunologically active [56] and are thought to participate in the immune-pathogenicity of paracoccidioido-mycosis [55], this might have an implication since *Paracoccidioides* cells are melanized during parasitism [13].

Another relevant protein observed in the melanized *Pb*18 proteome was the 43-kDa glycoprotein (PADG_07615), also known as gp43. This protein is immunogenic and can bind to laminin, presenting an important role in the pathogenesis of paracoccidioido-mycosis [9]. The strain *Pb*18 is known to produce this antigen [57], and this work has shown that L-DOPA may enhance the production of this protein. Contrastingly, the closest *P. lutzii* ortholog to gp43 did not present differential abundance in our analyses, which could be associated to the protein’s low expression by this species [58]. Here again, another difference related to L-DOPA’s influence was demonstrated between the two strains studied. Moreover, the differences in the abundance of this antigen may be related to the frequent false-negative results in patients with paracoccidioido-mycosis caused by *P. lutzii* frequently yield when gp43 is used in serologic tests [7].

In addition, *P. brasiliensis Pb*18 presented two abundant superoxide dismutases in melanized cells. These enzymes are involved in the primary detoxification defense against reactive oxygen species and, in *P. brasiliensis* phylogenetic species PS3 (or *P. restrepiensis*), at least superoxide dismutase isoform 3 is required for virulence [59]. This isoform did not present differential abundance in our proteomic analysis, but the role of those two *Pb*18 superoxide dismutase isoforms detected in *Paracoccidioides* virulence has not been properly addressed so far. Interestingly, *P. lutzii Pb*01 presented one superoxide dismutase (isoform 4) exclusively detected in non-melanized cells, suggesting that the melanin produced by this species, when L-DOPA is available during fungal growth, may be more efficient than *Pb*18 melanin in countering reactive oxygen species.

## 5. Conclusions

*Paracoccidioides* spp. respond to L-DOPA availability during growth by producing melanin, changing several metabolic pathways, and expressing virulence-related proteins. Although similar, the responses of the two studied species, *P. brasiliensis* and *P. lutzii*, to L-DOPA have some differences, such as a deeper change in protein abundance and metabolic pathways in *P. brasiliensis*, enhanced production of the immunodominant antigen in melanized *P. brasiliensis*, and lower abundance of proteins associated with detoxification of reactive oxygen species, such as those involved in peroxisome and superoxide dismutase isoform 6, in melanized *P. lutzii*. Further studies are necessary to address if the melanin produced by these two species has, indeed, biophysical differences that may be associated with the proteomic differences regarding detoxification measures observed in the current study.

## Figures and Tables

**Table 1:** Enriched Kyoto Encyclopedia of Genes and Genomes (KEGG) pathways observed for proteins with differential abundance in proteomes of melanized and non-melanized cells of *Paracoccidioides brasiliensis* and *Paracoccidioides lutzii*.

Pathway	*Pb*18		*Pb*01	
	Count in Gene Set	False Discovery Rate	Count in Gene Set	False Discovery Rate
Carbon metabolism				
Glycolysis/Gluconeogenesis	13/34	1.41 × 10^−18^	4/33	7.21 × 10^−6^
Oxidative phosphorylation	13/71	4.64 × 10^−15^	Not enriched	
Citrate cycle	9/27	1.66 × 10^−12^	4/26	3.70 × 10^−6^
Methane metabolism	8/20	1.03 × 10^−11^	7/19	1.21 × 10^−12^
Pentose phosphate pathway	8/23	2.33 × 10^−11^	5/23	3.75 × 10^−8^
Glyoxylate and dicarboxylate metabolism	7/33	8.81 × 10^−9^	5/21	1.24 × 10^−7^
Fatty Acid metabolism	3/29	0.0018	Not enriched	
Nucleotide metabolism				
Purine metabolism	27/86	6.77 × 10^−43^	11/80	7.10 × 10^−18^
Pyrimidine metabolism	19/61	4.25 × 10^−29^	11/56	2.94 × 10^−19^
Amino acid metabolism				
Val, Leu, and Ile degradation	13/28	1.19 × 10^−17^	6/26	7.04 × 10^−8^
Tyr metabolism	12/30	1.01 × 10^−15^	10/27	2.73 × 10^−14^
Ala, Asp, and Glu metabolism	11/30	3.49 × 10^−14^	7/30	4.21 × 10^−9^
Phe, Tyr, and Trp biosynthesis	7/17	1.32 × 10^−9^	5/16	2.82 × 10^−7^
Gly, Ser, and Thr metabolism	8/40	8.04 × 10^−9^	9/36	1.09 × 10^−11^
Phe metabolism	7/25	1.07 × 10^−8^	7/23	9.43 × 10^−10^
Val, Leu, and Ile biosynthesis	5/14	7.10 × 10^−7^	3/12	0.00017
Beta-Ala metabolism	5/19	2.39 × 10^−6^	2/16	0.0066
Lys degradation	4/19	5.97 × 10^−5^	4/18	1.84 × 10^−5^
Arg biosynthesis	4/19	5.97 × 10^−5^	3/19	0.00048
Other pathways				
Spliceosome	21/83	2.60 × 10^−17^	Not enriched	
RNA degradation	17/48	3.07 × 10^−16^	8/41	1.28 × 10^−10^
RNA transport	19/86	4.00 × 10^−15^	12/83	1.70 × 10^−14^
Protein processing in endoplasmic reticulum	18/74	4.45 × 10^−15^	Not enriched	
Phagosome	14/35	2.51 × 10^−14^	Not enriched	
Aminoacyl t-RNA biosynthesis	13/38	1.10 × 10^−12^	Not enriched	
Proteasome	11/35	1.59 × 10^−10^	Not enriched	
Basal transcription factors	10/29	5.35 × 10^−10^	Not enriched	
SNARE interactions in vesicular transport	3/15	0.0080	Not enriched	
Ubiquitin mediated proteolysis	4/52	0.0299	Not enriched	
mRNA surveillance	Not enriched		8/44	1.41 × 10^−10^
Peroxisome	Not enriched		8/51	3.06 × 10^−10^

Ala–Alanine; Arg–Arginine; Asp–Aspartate; Glu–Glutamate; Gly–Glycine; Ile–Isoleucine; Leu–Leucine; Lys–Lysine; Phe–Phenylalanine; Ser–Serine; Thr–Threonine; Trp–Tryptophan; Tyr–Tyrosine; Val–Valine; SNARE–Soluble N-ethylmaleimide-sensitive-fusion attachment protein receptor.

## Supplementary Materials

The following are available online at https://www.mdpi.com/2309-608X/6/4/328/s1, Figure S1: Electrophoretic profile of the protein extracts from melanized and non-melanized cells of *Paracoccidioides brasiliensis Pb*18 and *Paracoccidioides lutzii Pb*01. Figure S2: Venn diagrams presenting the relationship between the proteome of melanized and non-melanized cells of *Paracoccidioides brasiliensis Pb*18 and *Paracoccidioides lutzii Pb*01. Figure S3: Protein-protein interaction networks of the differentially abundant proteins of *Paracoccidiodies brasiliensis Pb*18 and *Paracoccidioides lutzii Pb*01. Figure S4: Enriched KEGG pathways associated with carbon metabolism in the proteomes of melanized *Paracoccidioides* species. (**a**) protein-protein interactions of 49 *P. brasiliensis Pb*18 proteins; (**b**) protein-protein interactions of 24 *P. lutzii Pb*01 proteins. Figure S5: Enriched KEGG pathways associated with nucleotide metabolism in the proteomes of melanized *Paracoccidioides* species. (**a**) protein-protein interactions of 37 *P. brasiliensis Pb*18 proteins; (**b**) protein-protein interactions of 18 *P. lutzii Pb*01 proteins. Figure S6: Enriched KEGG pathways associated with amino acid metabolism in the proteomes of melanized *Paracoccidioides* species. (**a**) protein-protein interactions of 49 *P. brasiliensis Pb*18 proteins; (**b**) protein-protein interactions of 24 *P. lutzii Pb*01 proteins. Figure S7: Other enriched KEGG pathways in the proteomes of melanized *Paracoccidioides* species. (**a**) protein-protein interactions of 123 *P. brasiliensis Pb*18 proteins from 10 different pathways; (**b**) protein-protein interactions of 35 *P. lutzii Pb*01 proteins from four different pathways. Figure S8: Comparison of the abundance in melanized cells of proteins associated with enriched pathways observed on proteomes of both *Paracoccidiodies brasiliensis Pb*18 and *Paracoccidiodies lutzii Pb*01. Figure S9: Interaction between nucleotide and amino acid metabolisms with the tricarboxylic acid and glyoxylate cycles. Amino acids in red represent potential carbon sources for the tricarboxylic acid cycle in melanized *Paracoccidioides* spp. cells. Table S1: Proteins with differential abundance in *Paracoccidioides brasiliensis Pb*18 proteome and their classification after Benjamini–Hochberg *q*-value, F-stringency, and L-stringency analyses. Table S2: Proteins with differential abundance in *Paracoccidioides lutzii Pb*01 proteome and their classification after Benjamini–Hochberg *q*-value, F-stringency, and L-stringency analyses.
