# Supplementary material for: Beyond Melanin: Proteomics Reveals Virulence-Related Proteins in Paracoccidioides brasiliensis and Paracoccidioides lutzii Yeast Cells Grown in the Presence of L-Dihydroxyphenylalanine
Source: J Fungi (Basel). 2020 Dec 1;6(4):328. doi: 10.3390/jof6040328 (PMC7711940; doi:10.3390/jof6040328)
Supplement: Supplementary file 1 [file jof-06-00328-s001.zip › FigureS8.pdf]

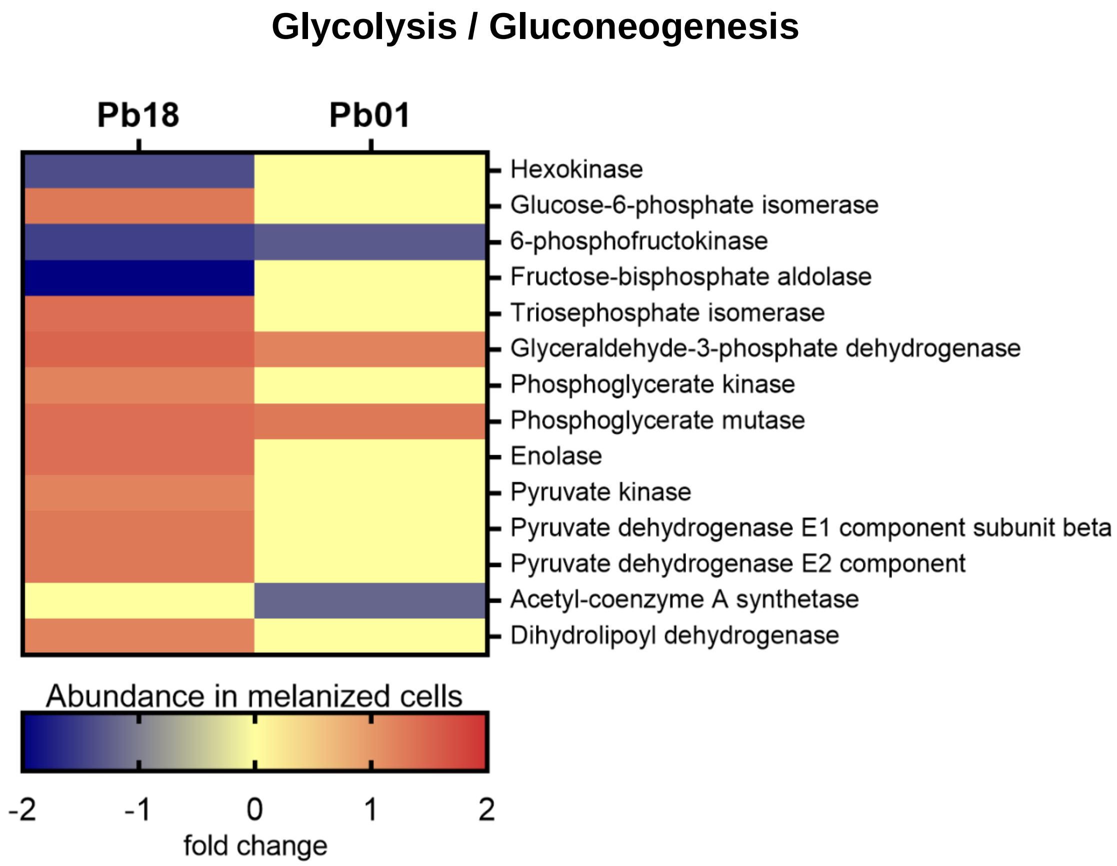
Glycolysis / Gluconeogenesis
Pb18
Pb01
Hexokinase
Glucose-6-phosphate isomerase
6-phosphofructokinase
Fructose-bisphosphate aldolase
Triosephosphate isomerase
Glyceraldehyde-3-phosphate dehydrogenase
Phosphoglycerate kinase
Phosphoglycerate mutase
Enolase
Pyruvate kinase
Pyruvate dehydrogenase E1 component subunit beta
Pyruvate dehydrogenase E2 component
Acetyl-coenzyme A synthetase
Dihydrolipoyl dehydrogenase
Abundance in melanized cells
-2
-1
0
1
2
fold change

Tricarboxylic acid cycle

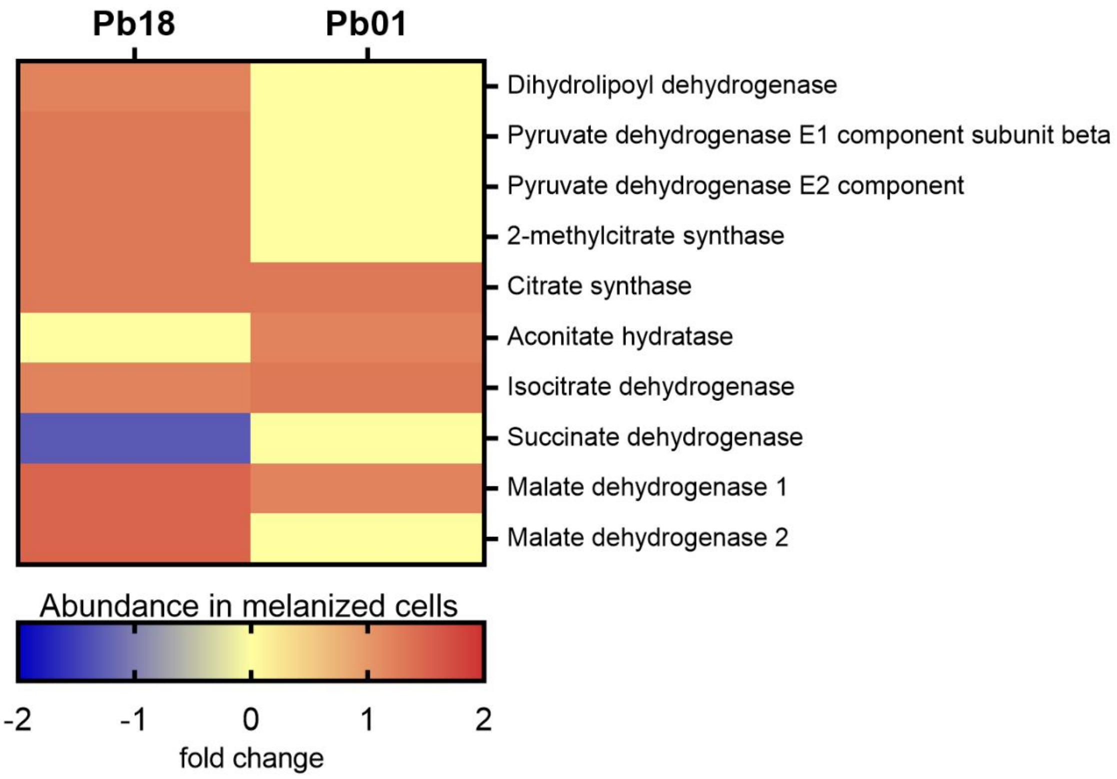

Glyoxylate cycle

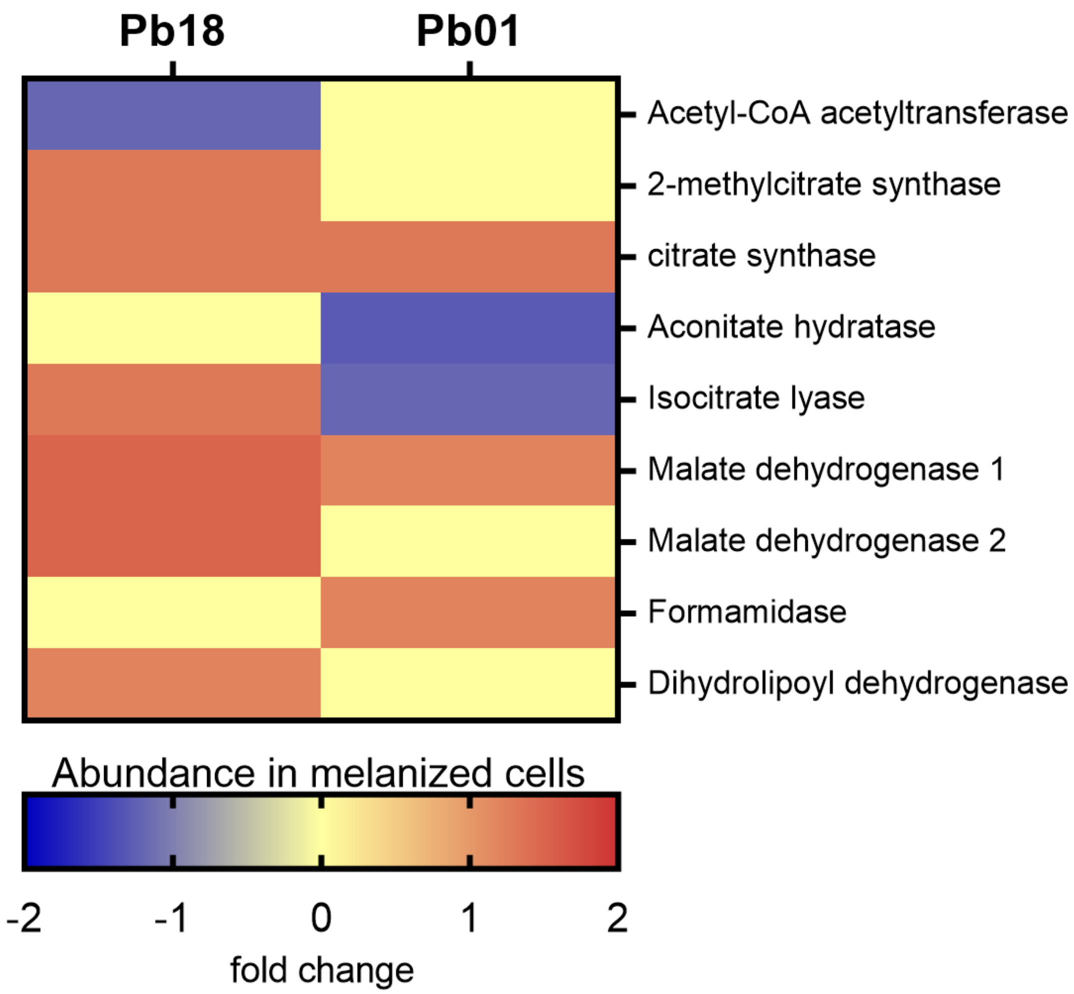

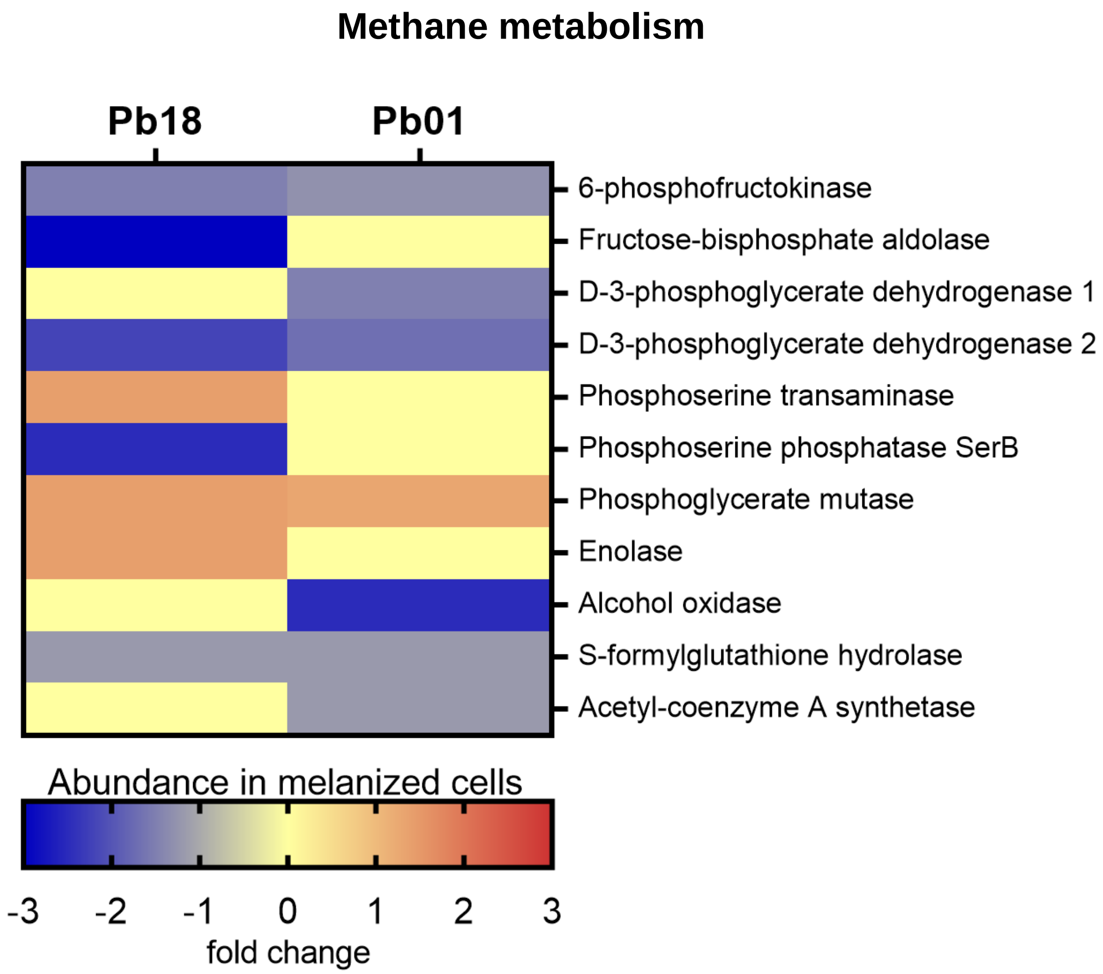

Methane metabolism
Pb18
Pb01
6-phosphofructokinase
Fructose-bisphosphate aldolase
D-3-phosphoglycerate dehydrogenase 1
D-3-phosphoglycerate dehydrogenase 2
Phosphoserine transaminase
Phosphoserine phosphatase SerB
Phosphoglycerate mutase
Enolase
Alcohol oxidase
S-formylglutathione hydrolase
Acetyl-coenzyme A synthetase
Abundance in melanized cells
-3
-2
-1
0
1
2
3
fold change

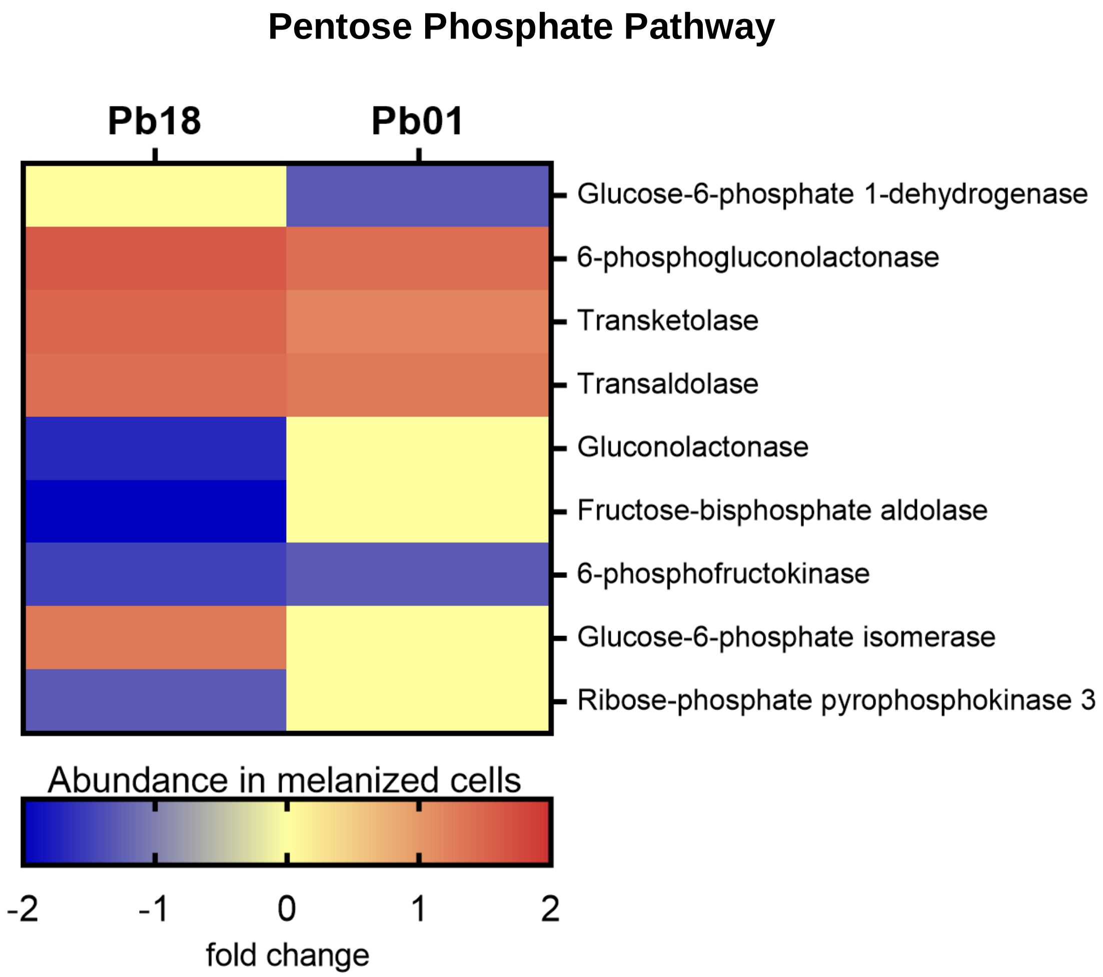
Pentose Phosphate Pathway
Pb18
Pb01
Glucose-6-phosphate 1-dehydrogenase
6-phosphogluconolactonase
Transketolase
Transaldolase
Gluconolactonase
Fructose-bisphosphate aldolase
6-phosphofructokinase
Glucose-6-phosphate isomerase
Ribose-phosphate pyrophosphokinase 3
Abundance in melanized cells
-2
-1
0
1
2
fold change

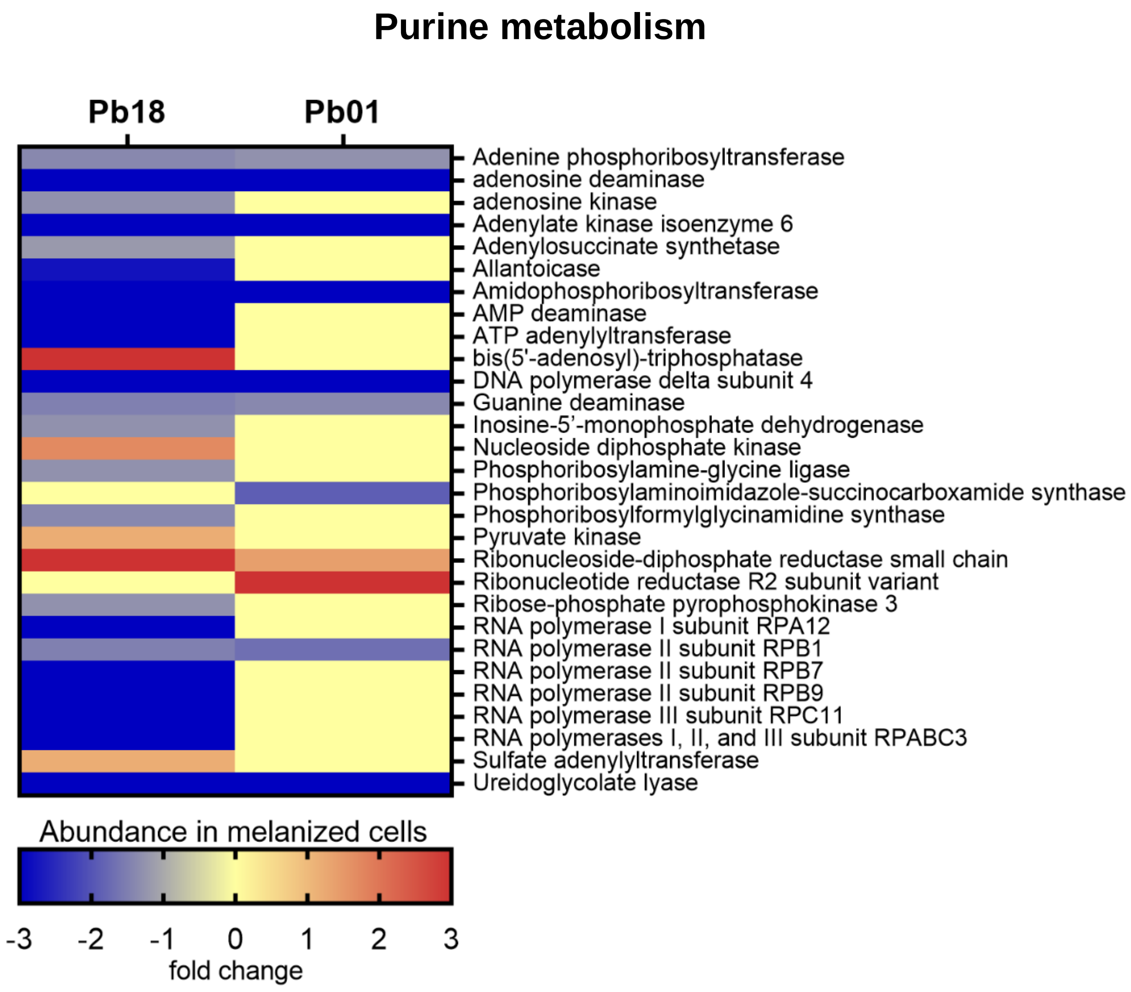
Purine metabolism
Pb18
Pb01
Adenine phosphoribosyltransferase
adenosine deaminase
adenosine kinase
Adenylate kinase isoenzyme 6
Adenylosuccinate synthetase
Allantoicase
Amidophosphoribosyltransferase
AMP deaminase
ATP adenylyltransferase
bis(5'-adenosyl)-triphosphatase
DNA polymerase delta subunit 4
Guanine deaminase
Inosine-5'-monophosphate dehydrogenase
Nucleoside diphosphate kinase
Phosphoribosylamine-glycine ligase
Phosphoribosylaminoimidazole-succinocarboxamide synthase
Phosphoribosylformylglycinamidine synthase
Pyruvate kinase
Ribonucleoside-diphosphate reductase small chain
Ribonucleotide reductase R2 subunit variant
Ribose-phosphate pyrophosphokinase 3
RNA polymerase I subunit RPA12
RNA polymerase II subunit RPB1
RNA polymerase II subunit RPB7
RNA polymerase II subunit RPB9
RNA polymerase III subunit RPC11
RNA polymerases I, II, and III subunit RPABC3
Sulfate adenylyltransferase
Ureidoglycolate lyase
Abundance in melanized cells
-3
-2
-1
0
1
2
3
fold change

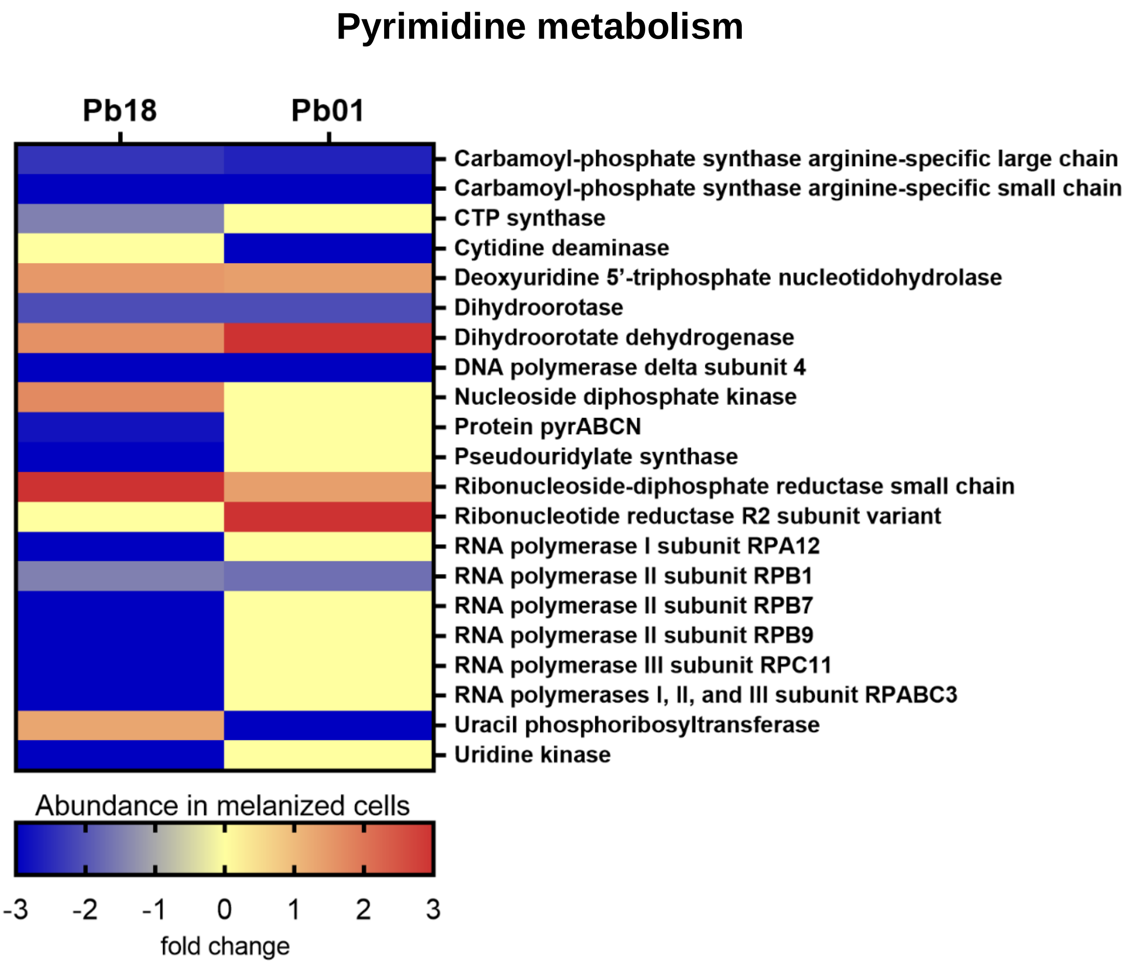

Pyrimidine metabolism
Pb18
Pb01
Carbamoyl-phosphate synthase arginine-specific large chain
Carbamoyl-phosphate synthase arginine-specific small chain
CTP synthase
Cytidine deaminase
Deoxyuridine 5'-triphosphate nucleotidohydrolase
Dihydroorotase
Dihydroorotate dehydrogenase
DNA polymerase delta subunit 4
Nucleoside diphosphate kinase
Protein pyrABCN
Pseudouridylate synthase
Ribonucleoside-diphosphate reductase small chain
Ribonucleotide reductase R2 subunit variant
RNA polymerase I subunit RPA12
RNA polymerase II subunit RPB1
RNA polymerase II subunit RPB7
RNA polymerase II subunit RPB9
RNA polymerase III subunit RPC11
RNA polymerases I, II, and III subunit RPABC3
Uracil phosphoribosyltransferase
Uridine kinase
Abundance in melanized cells
-3
-2
-1
0
1
2
3
fold change

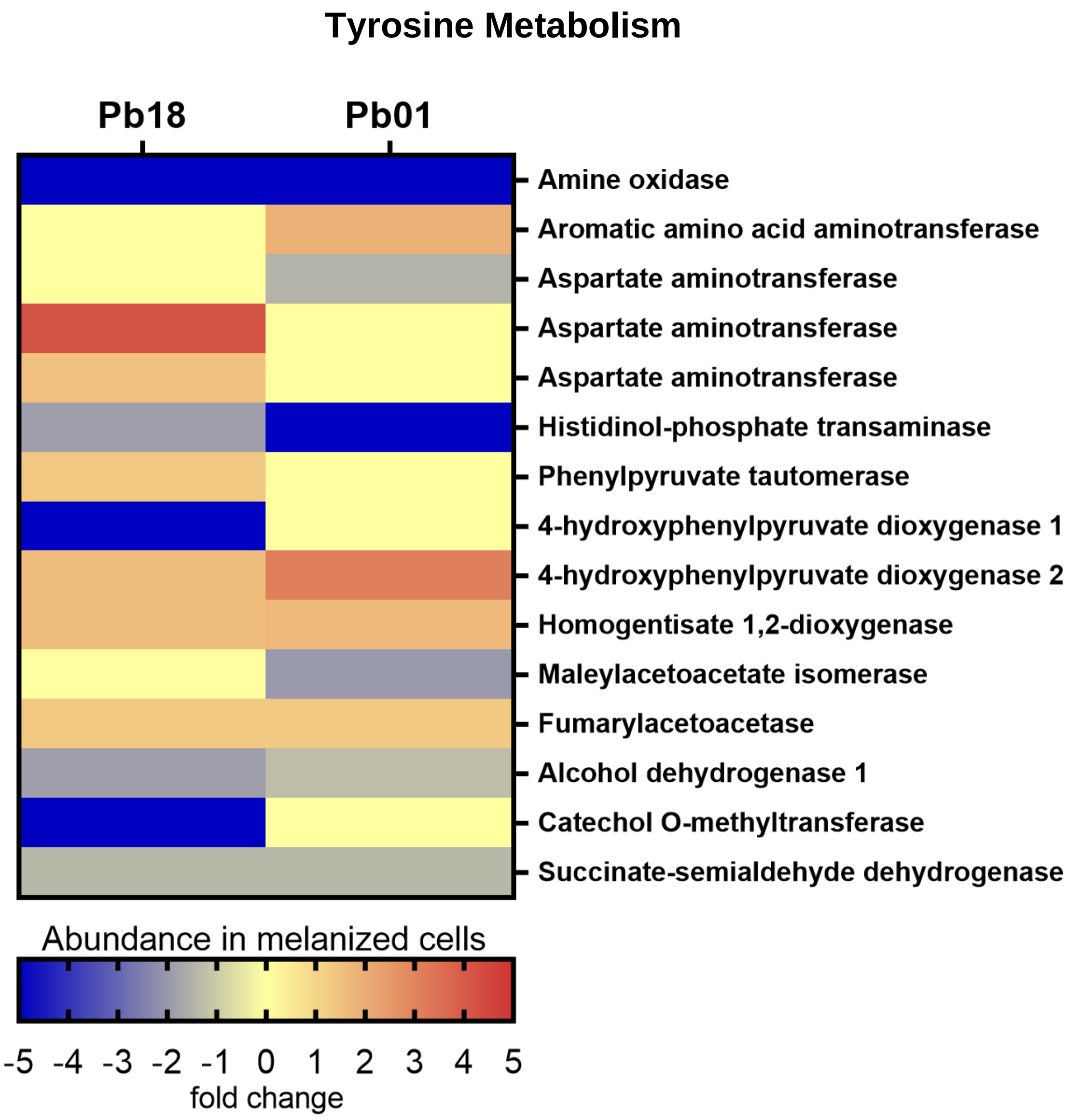
Tyrosine Metabolism
Pb18
Pb01
Amine oxidase
Aromatic amino acid aminotransferase
Aspartate aminotransferase
Aspartate aminotransferase
Aspartate aminotransferase
Histidinol-phosphate transaminase
Phenylpyruvate tautomerase
4-hydroxyphenylpyruvate dioxygenase 1
4-hydroxyphenylpyruvate dioxygenase 2
Homogentisate 1,2-dioxygenase
Maleylacetoacetate isomerase
Fumarylacetoacetase
Alcohol dehydrogenase 1
Catechol O-methyltransferase
Succinate-semialdehyde dehydrogenase
Abundance in melanized cells
-5 -4 -3 -2 -1 0 1 2 3 4 5
fold change

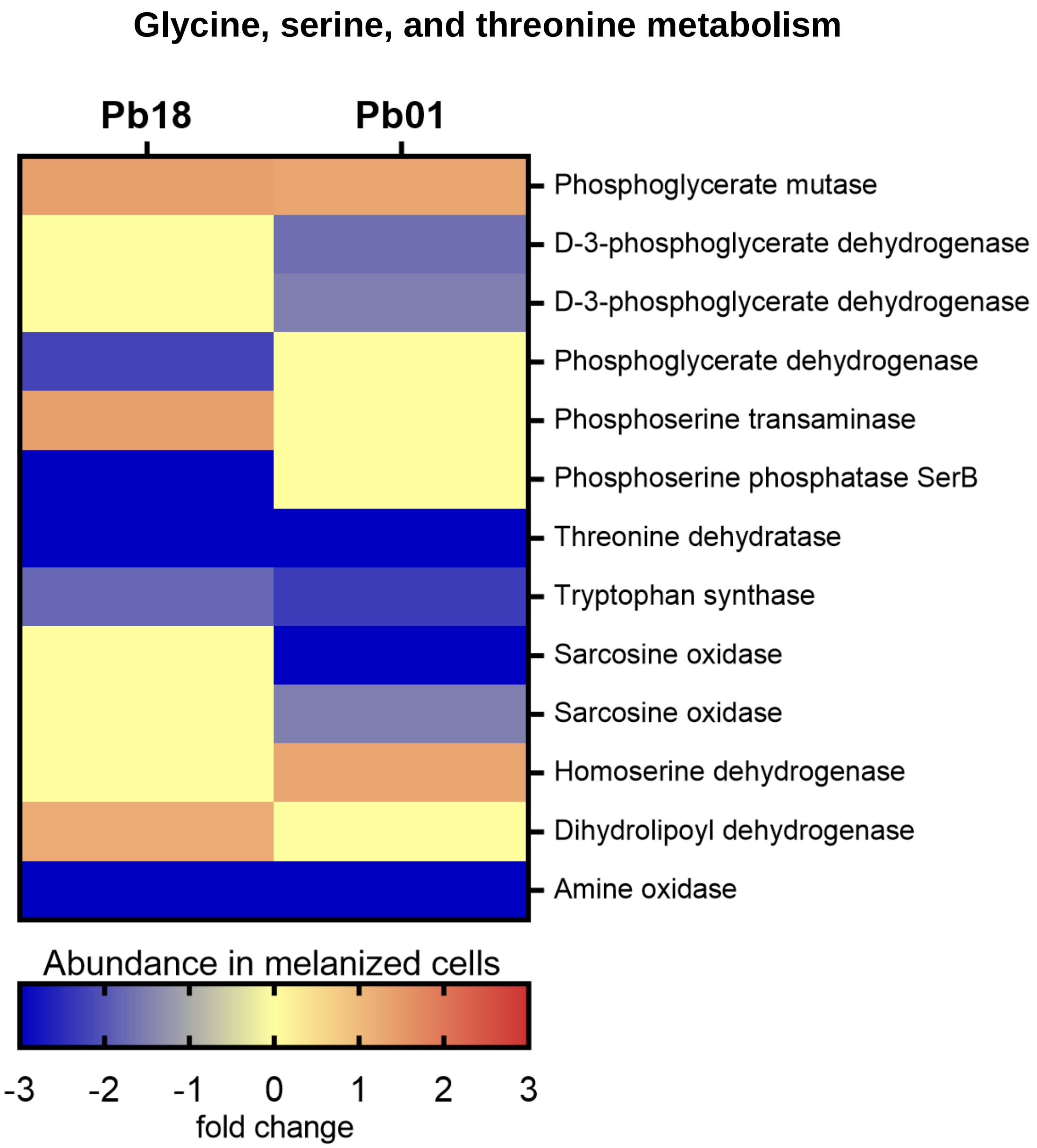

Glycine, serine, and threonine metabolism
Pb18
Pb01
Phosphoglycerate mutase
D-3-phosphoglycerate dehydrogenase
D-3-phosphoglycerate dehydrogenase
Phosphoglycerate dehydrogenase
Phosphoserine transaminase
Phosphoserine phosphatase SerB
Threonine dehydratase
Tryptophan synthase
Sarcosine oxidase
Sarcosine oxidase
Homoserine dehydrogenase
Dihydrolipoyl dehydrogenase
Amine oxidase
Abundance in melanized cells
-3 -2 -1 0 1 2 3
fold change

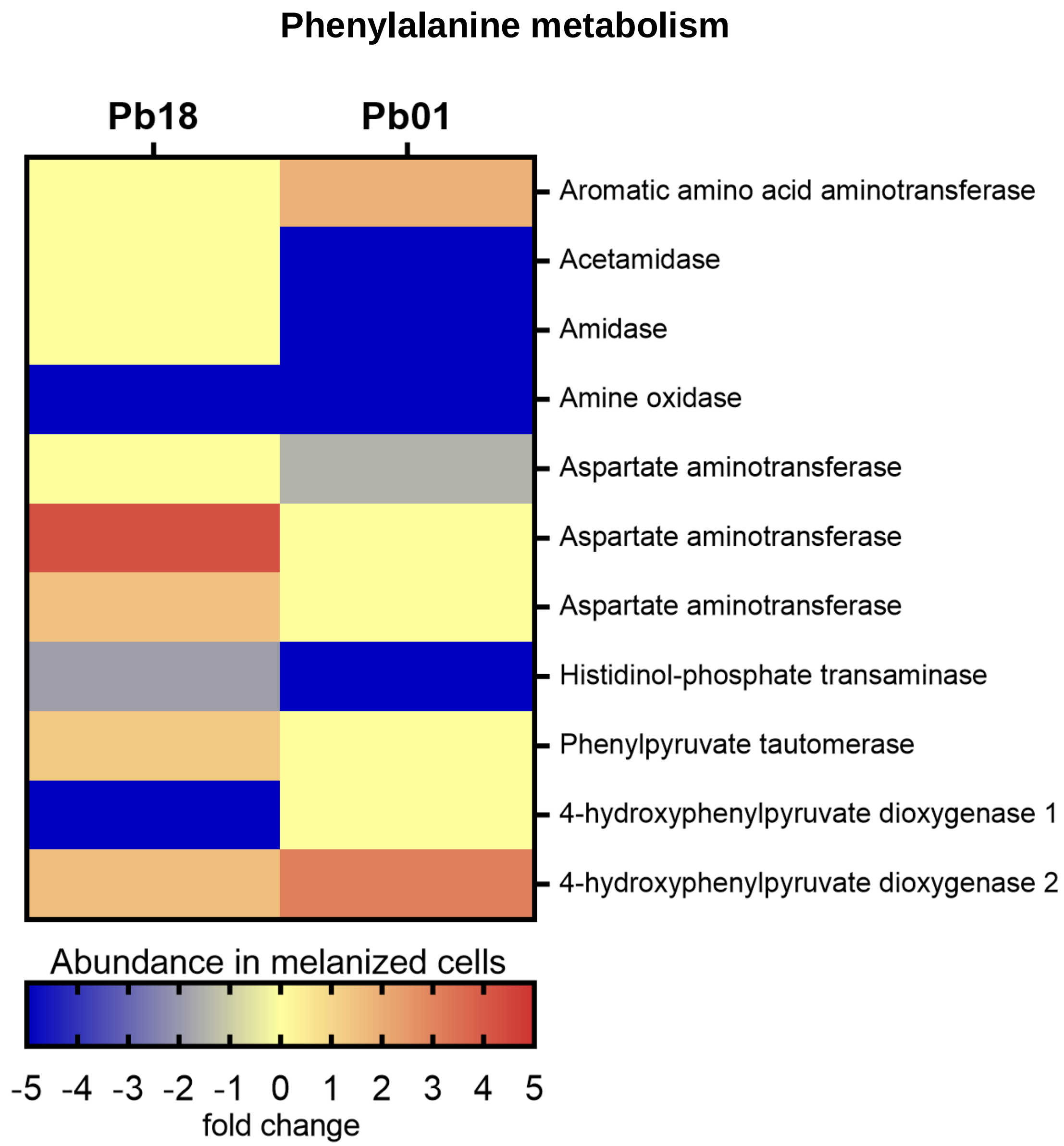
Phenylalanine metabolism
Pb18
Pb01
Aromatic amino acid aminotransferase
Acetamidase
Amidase
Amine oxidase
Aspartate aminotransferase
Aspartate aminotransferase
Aspartate aminotransferase
Histidinol-phosphate transaminase
Phenylpyruvate tautomerase
4-hydroxyphenylpyruvate dioxygenase 1
4-hydroxyphenylpyruvate dioxygenase 2
Abundance in melanized cells
-5 -4 -3 -2 -1 0 1 2 3 4 5
fold change

Alanine, aspartate, and glutamate metabolism

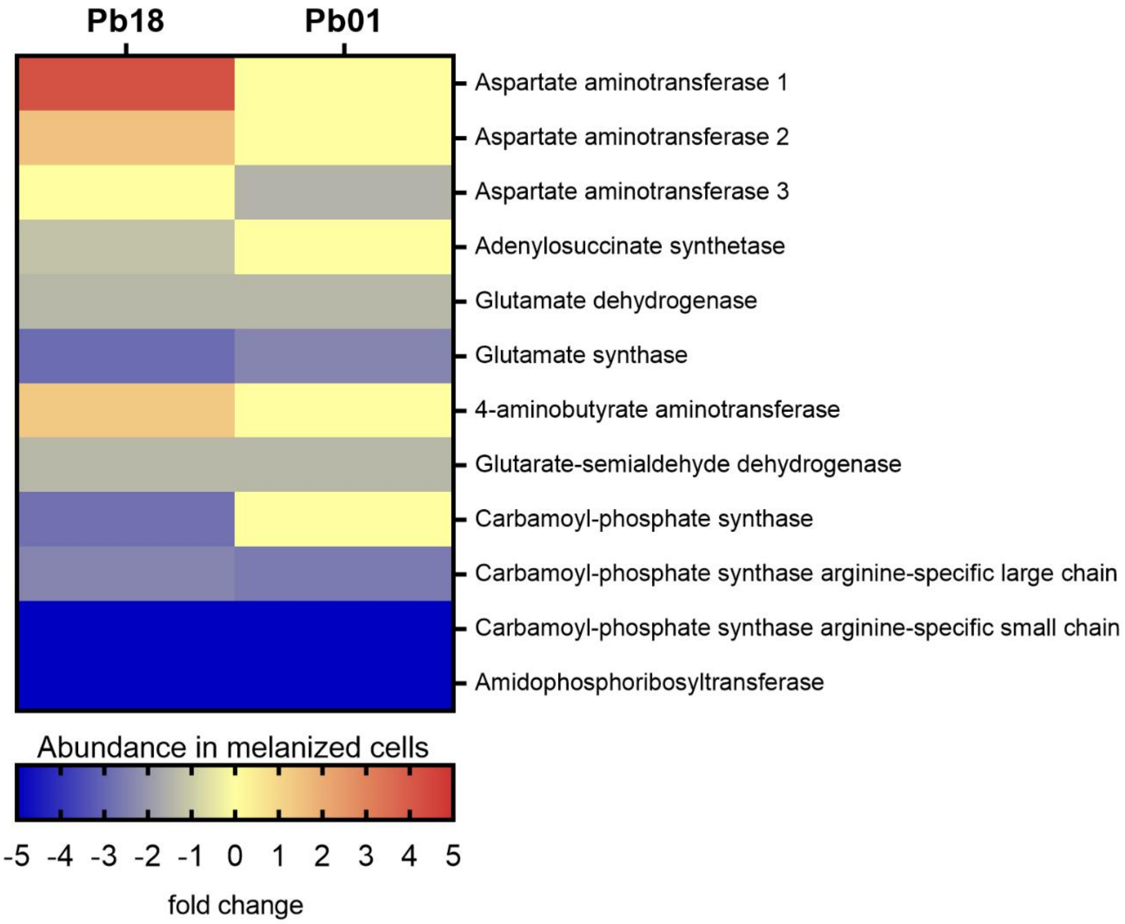

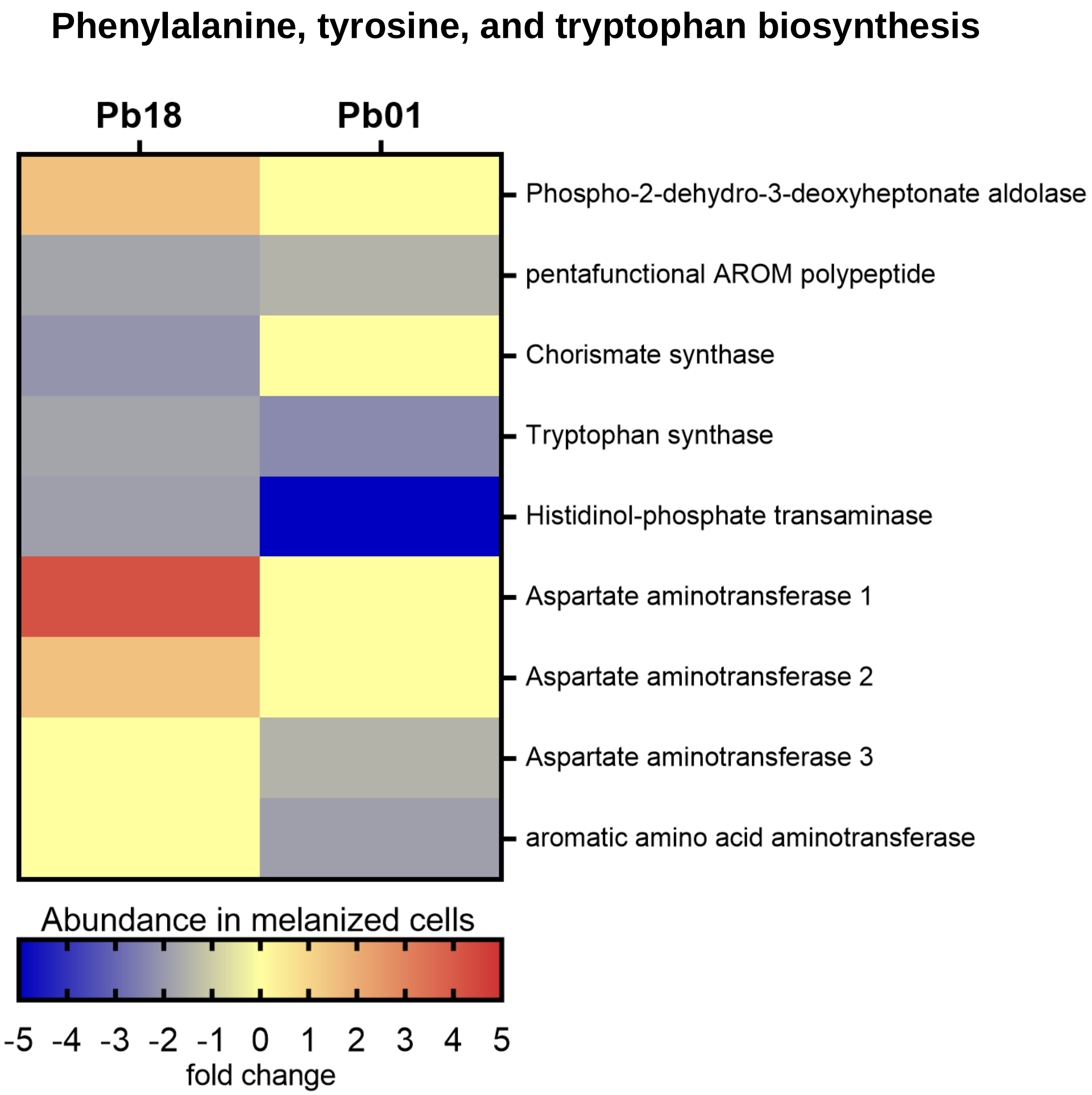

Phenylalanine, tyrosine, and tryptophan biosynthesis
Pb18
Pb01
Phospho-2-dehydro-3-deoxyheptonate aldolase
pentafunctional AROM polypeptide
Chorismate synthase
Tryptophan synthase
Histidinol-phosphate transaminase
Aspartate aminotransferase 1
Aspartate aminotransferase 2
Aspartate aminotransferase 3
aromatic amino acid aminotransferase
Abundance in melanized cells
-5 -4 -3 -2 -1 0 1 2 3 4 5
fold change

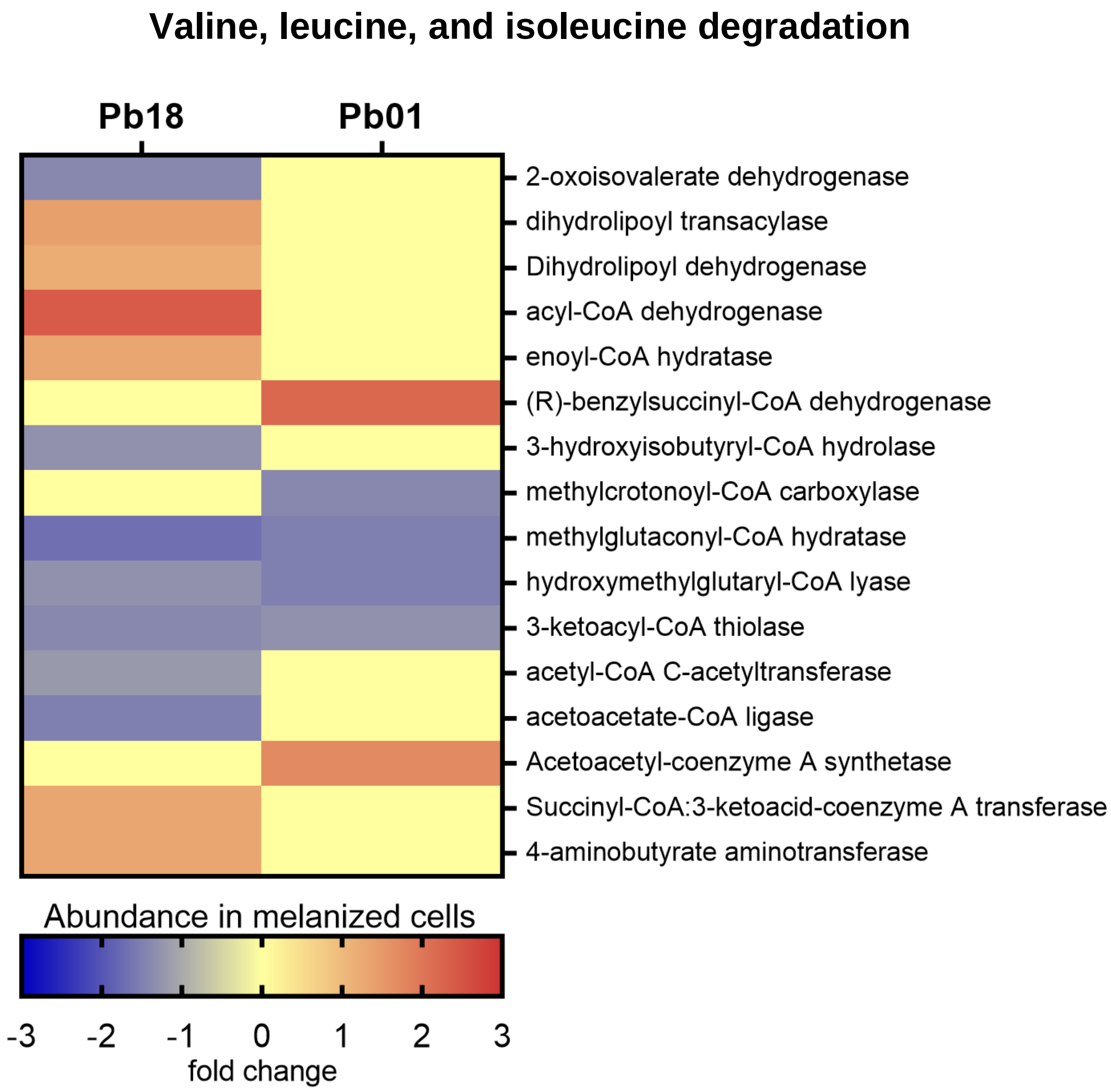
Valine, leucine, and isoleucine degradation
Pb18
Pb01
2-oxoisovalerate dehydrogenase
dihydrolipoyl transacylase
Dihydrolipoyl dehydrogenase
acyl-CoA dehydrogenase
enoyl-CoA hydratase
(R)-benzylsuccinyl-CoA dehydrogenase
3-hydroxyisobutyryl-CoA hydrolase
methylcrotonoyl-CoA carboxylase
methylglutaconyl-CoA hydratase
hydroxymethylglutaryl-CoA lyase
3-ketoacyl-CoA thiolase
acetyl-CoA C-acetyltransferase
acetoacetate-CoA ligase
Acetoacetyl-coenzyme A synthetase
Succinyl-CoA:3-ketoacid-coenzyme A transferase
4-aminobutyrate aminotransferase
Abundance in melanized cells
-3
-2
-1
0
1
2
3
fold change

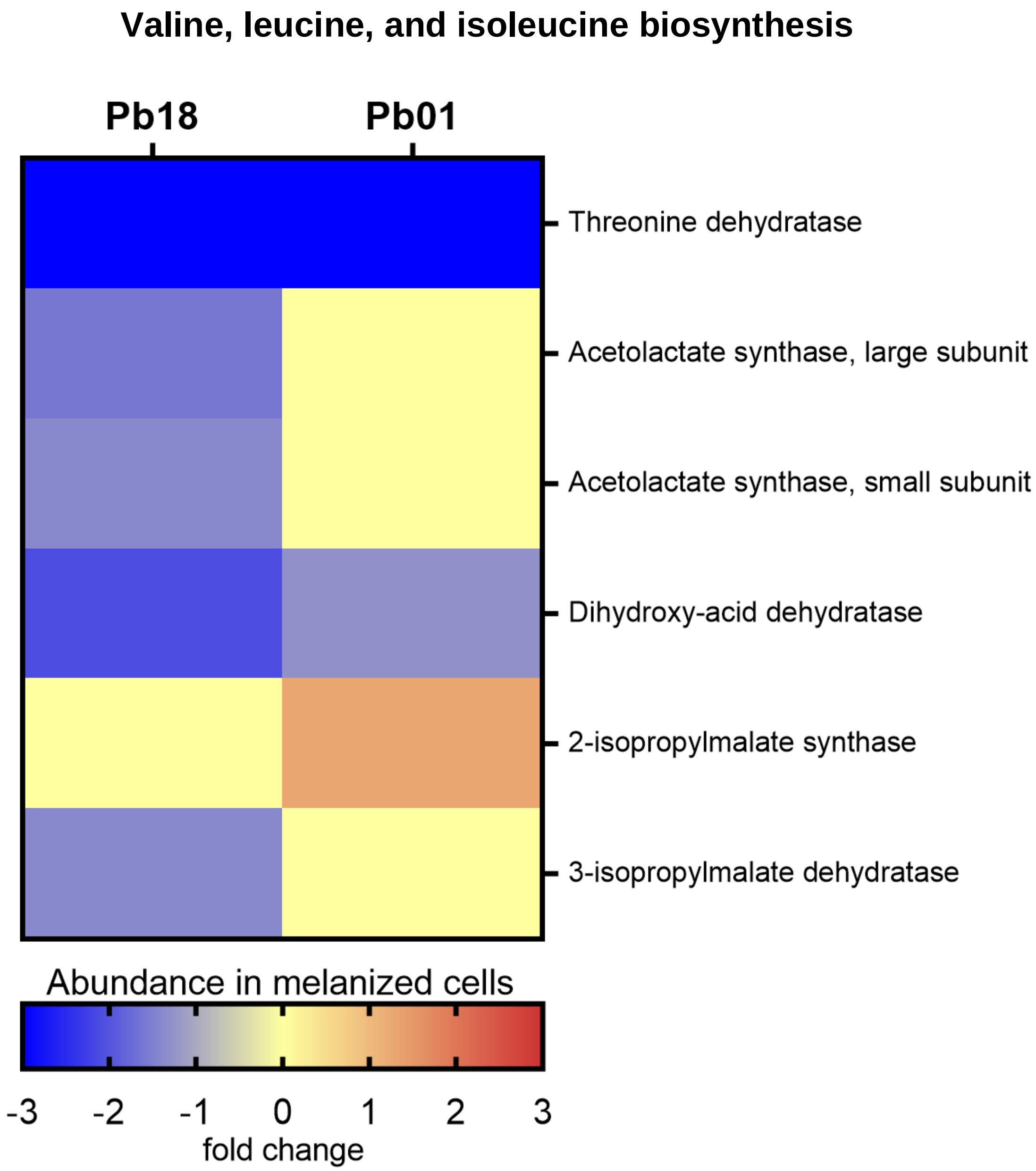
Valine, leucine, and isoleucine biosynthesis
Pb18
Pb01
Threonine dehydratase
Acetolactate synthase, large subunit
Acetolactate synthase, small subunit
Dihydroxy-acid dehydratase
2-isopropylmalate synthase
3-isopropylmalate dehydratase
Abundance in melanized cells
-3
-2
-1
0
1
2
3
fold change

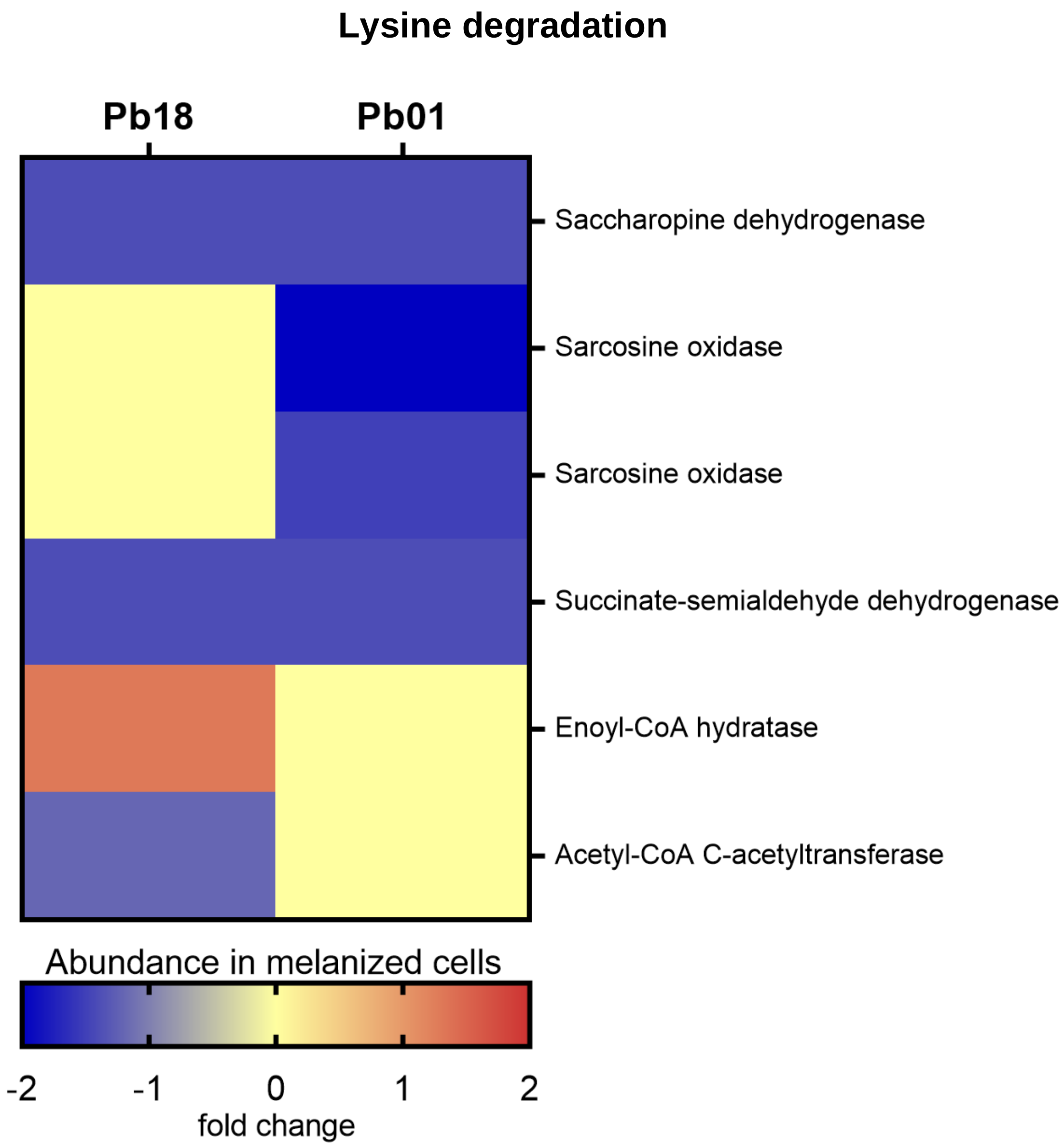

Lysine degradation
Pb18
Pb01
Saccharopine dehydrogenase
Sarcosine oxidase
Sarcosine oxidase
Succinate-semialdehyde dehydrogenase
Enoyl-CoA hydratase
Acetyl-CoA C-acetyltransferase
Abundance in melanized cells
-2
-1
0
1
2
fold change

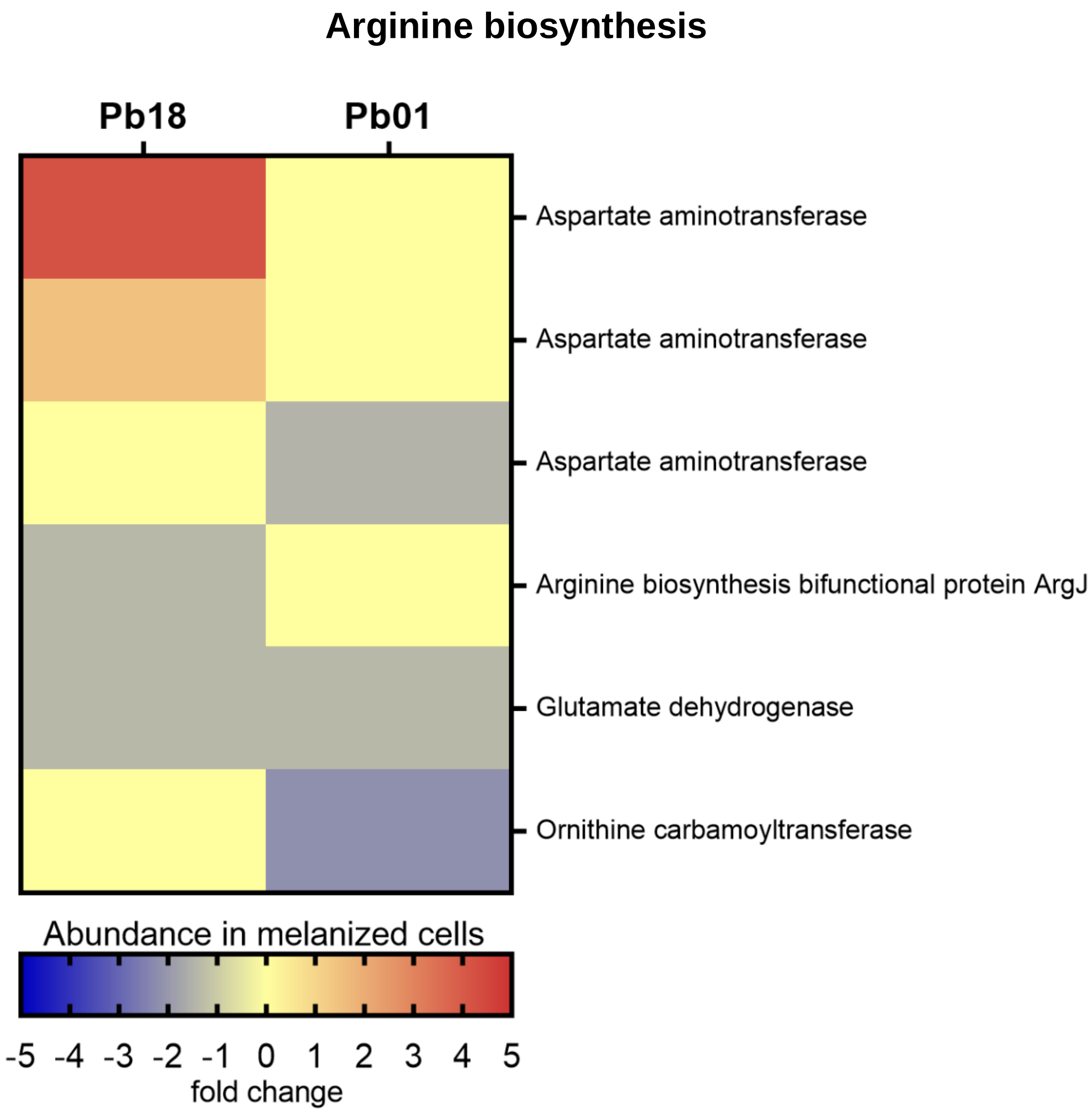

Arginine biosynthesis
Pb18
Pb01
Aspartate aminotransferase
Aspartate aminotransferase
Aspartate aminotransferase
Arginine biosynthesis bifunctional protein ArgJ
Glutamate dehydrogenase
Ornithine carbamoyltransferase
Abundance in melanized cells
-5 -4 -3 -2 -1 0 1 2 3 4 5
fold change

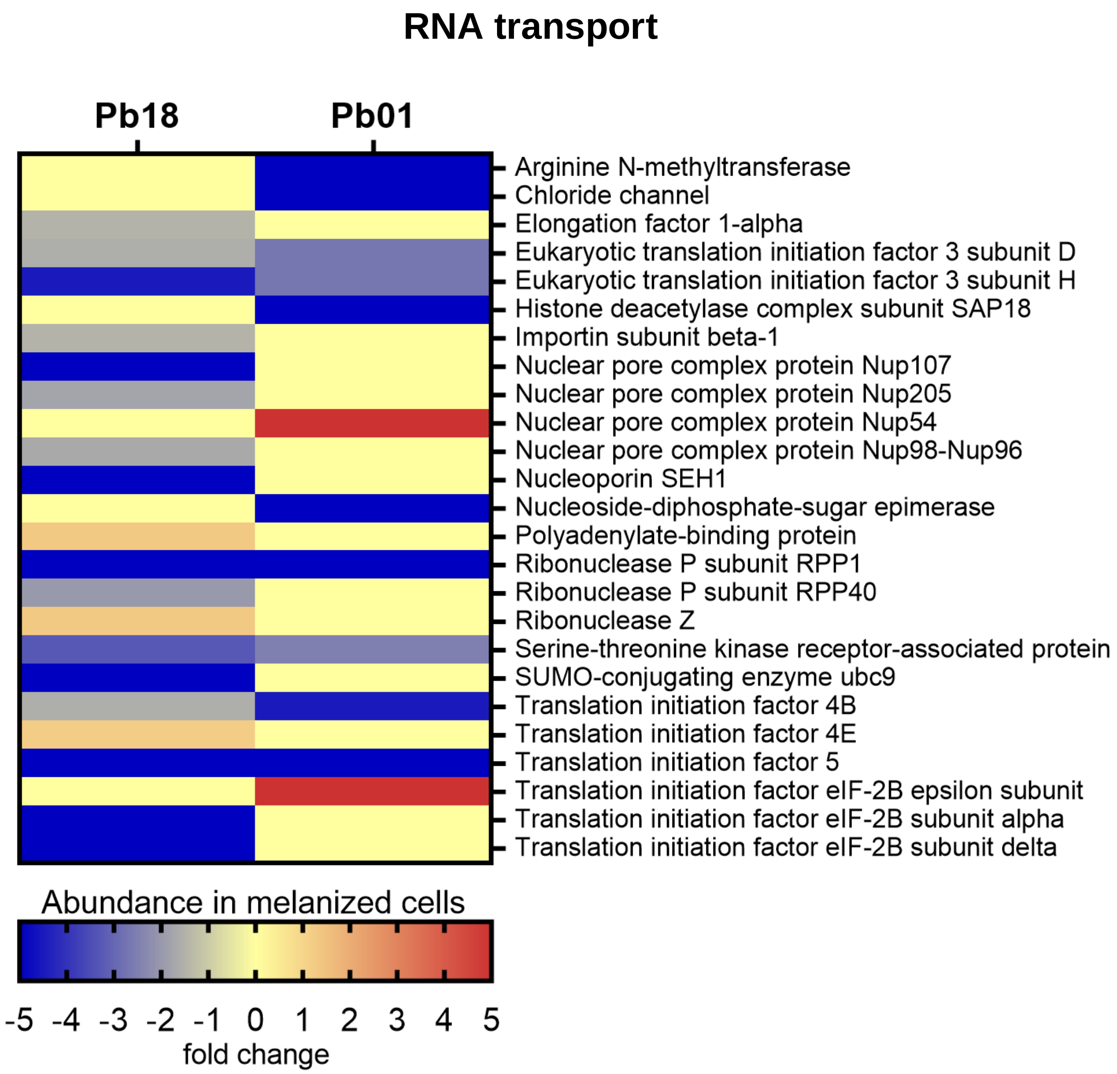
RNA transport
Pb18
Pb01
Arginine N-methyltransferase
Chloride channel
Elongation factor 1-alpha
Eukaryotic translation initiation factor 3 subunit D
Eukaryotic translation initiation factor 3 subunit H
Histone deacetylase complex subunit SAP18
Importin subunit beta-1
Nuclear pore complex protein Nup107
Nuclear pore complex protein Nup205
Nuclear pore complex protein Nup54
Nuclear pore complex protein Nup98-Nup96
Nucleoporin SEH1
Nucleoside-diphosphate-sugar epimerase
Polyadenylate-binding protein
Ribonuclease P subunit RPP1
Ribonuclease P subunit RPP40
Ribonuclease Z
Serine-threonine kinase receptor-associated protein
SUMO-conjugating enzyme ubc9
Translation initiation factor 4B
Translation initiation factor 4E
Translation initiation factor 5
Translation initiation factor eIF-2B epsilon subunit
Translation initiation factor eIF-2B subunit alpha
Translation initiation factor eIF-2B subunit delta
Abundance in melanized cells
-5 -4 -3 -2 -1 0 1 2 3 4 5
fold change

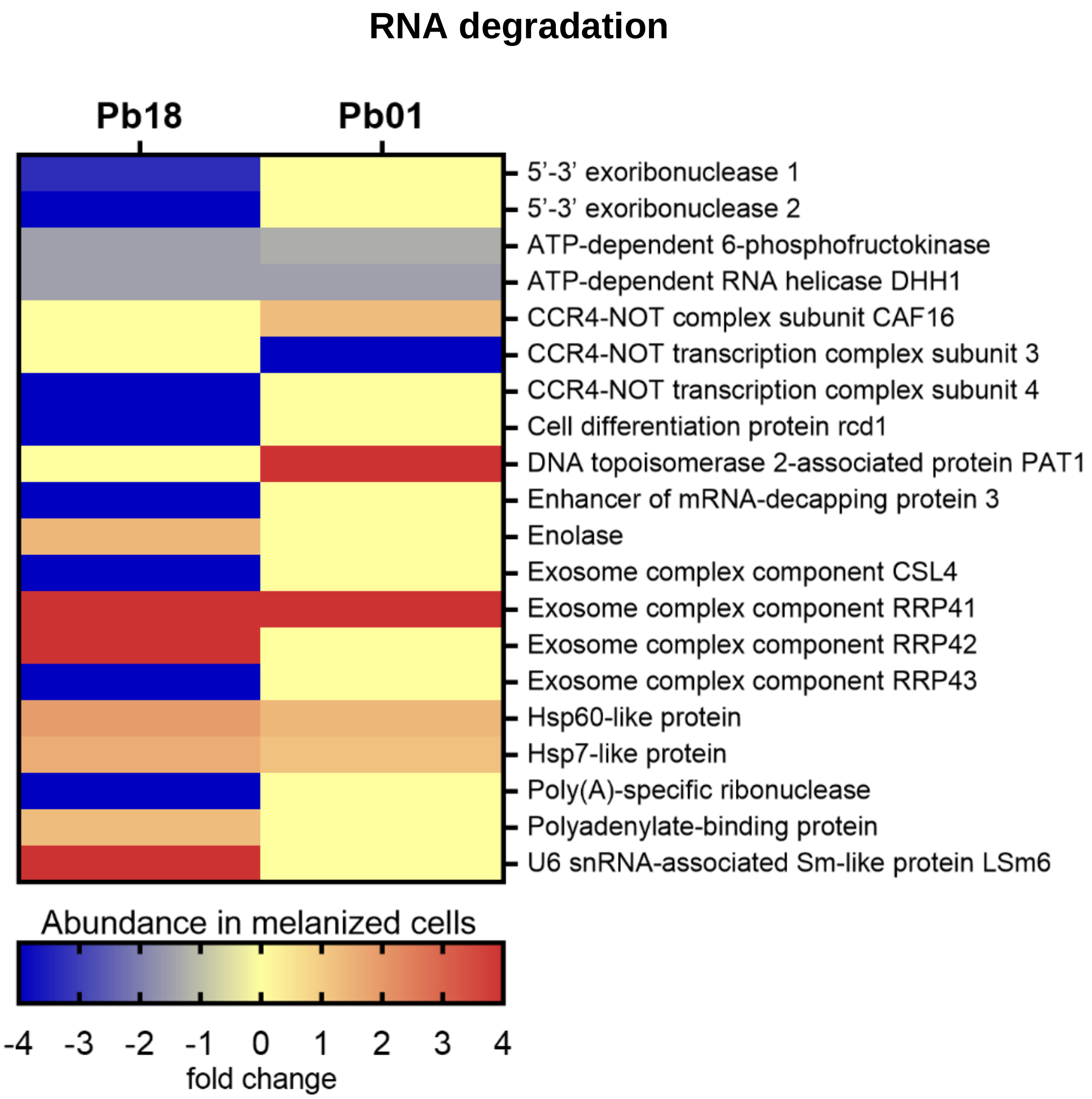
RNA degradation
Pb18
Pb01
5'-3' exoribonuclease 1
5'-3' exoribonuclease 2
ATP-dependent 6-phosphofructokinase
ATP-dependent RNA helicase DHH1
CCR4-NOT complex subunit CAF16
CCR4-NOT transcription complex subunit 3
CCR4-NOT transcription complex subunit 4
Cell differentiation protein rcd1
DNA topoisomerase 2-associated protein PAT1
Enhancer of mRNA-decapping protein 3
Enolase
Exosome complex component CSL4
Exosome complex component RRP41
Exosome complex component RRP42
Exosome complex component RRP43
Hsp60-like protein
Hsp7-like protein
Poly(A)-specific ribonuclease
Polyadenylate-binding protein
U6 snRNA-associated Sm-like protein LSm6
Abundance in melanized cells
-4
-3
-2
-1
0
1
2
3
4
fold change
