# Supplementary figures and images for: Beyond Melanin: Proteomics Reveals Virulence-Related Proteins in Paracoccidioides brasiliensis and Paracoccidioides lutzii Yeast Cells Grown in the Presence of L-Dihydroxyphenylalanine
Source: J Fungi (Basel). 2020 Dec 1;6(4):328. doi: 10.3390/jof6040328 (PMC7711940; doi:10.3390/jof6040328)

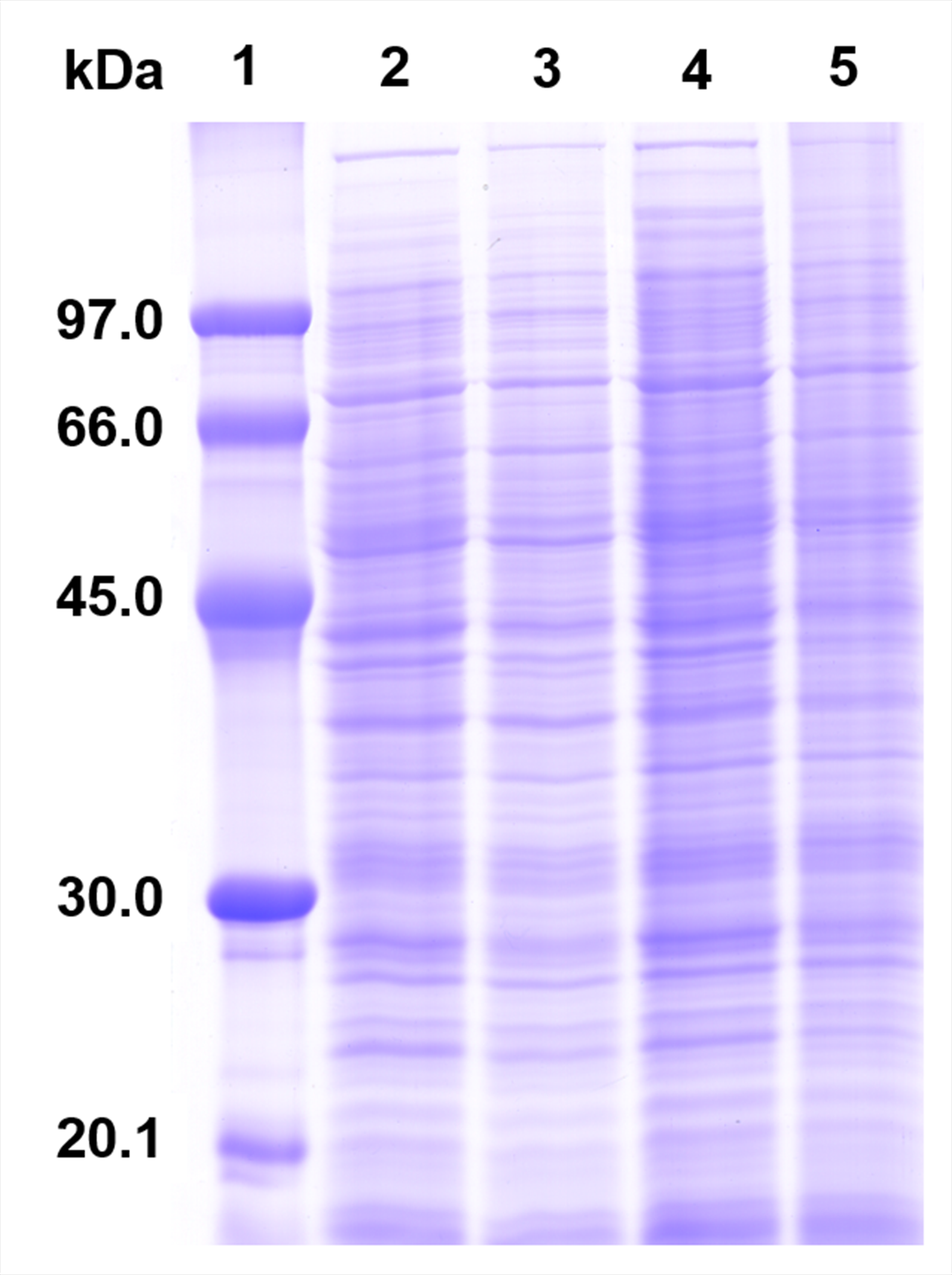

Supplement: Supplementary file 1 [file jof-06-00328-s001.zip › FigureS1.tif]

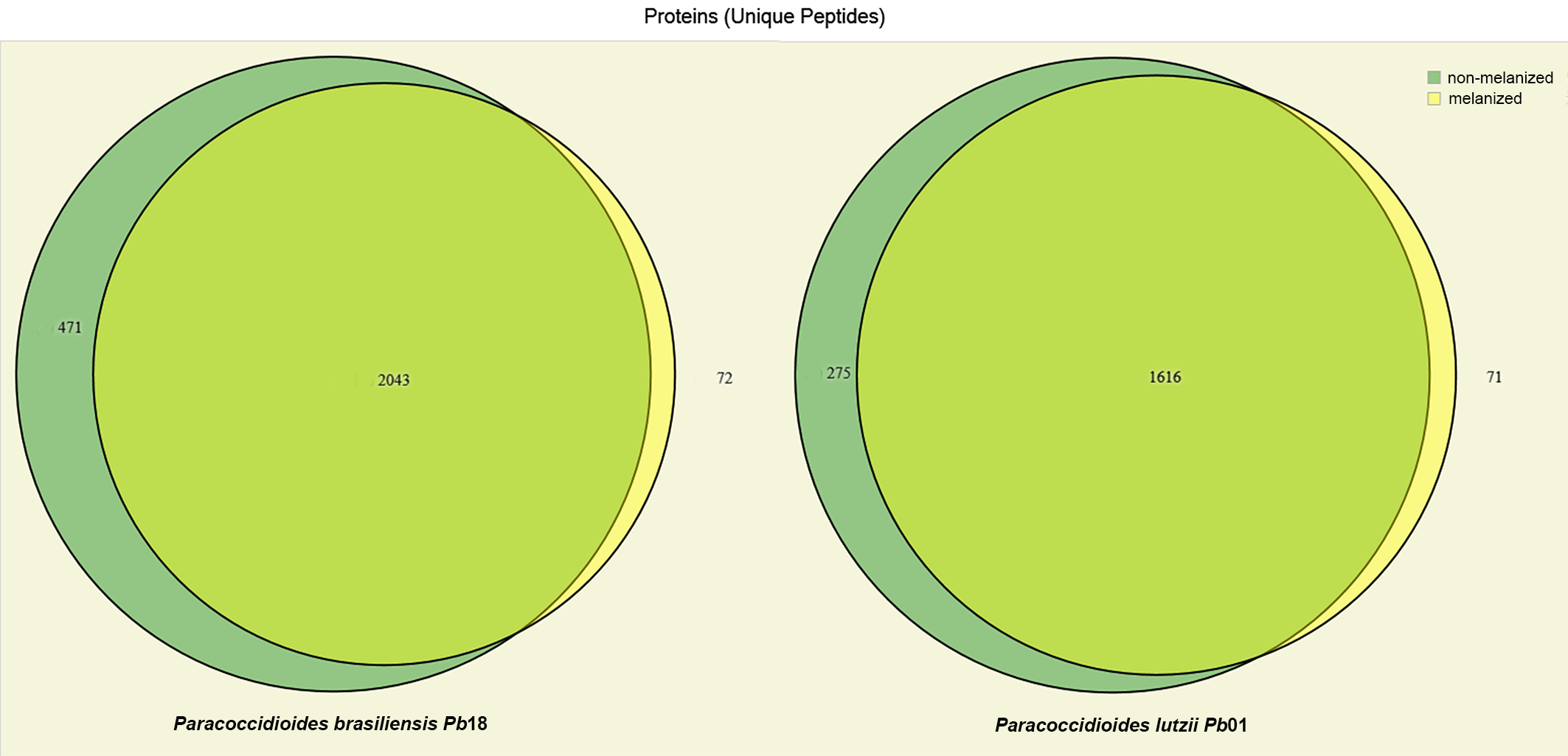

Supplement: Supplementary file 1 [file jof-06-00328-s001.zip › FigureS2.tif]

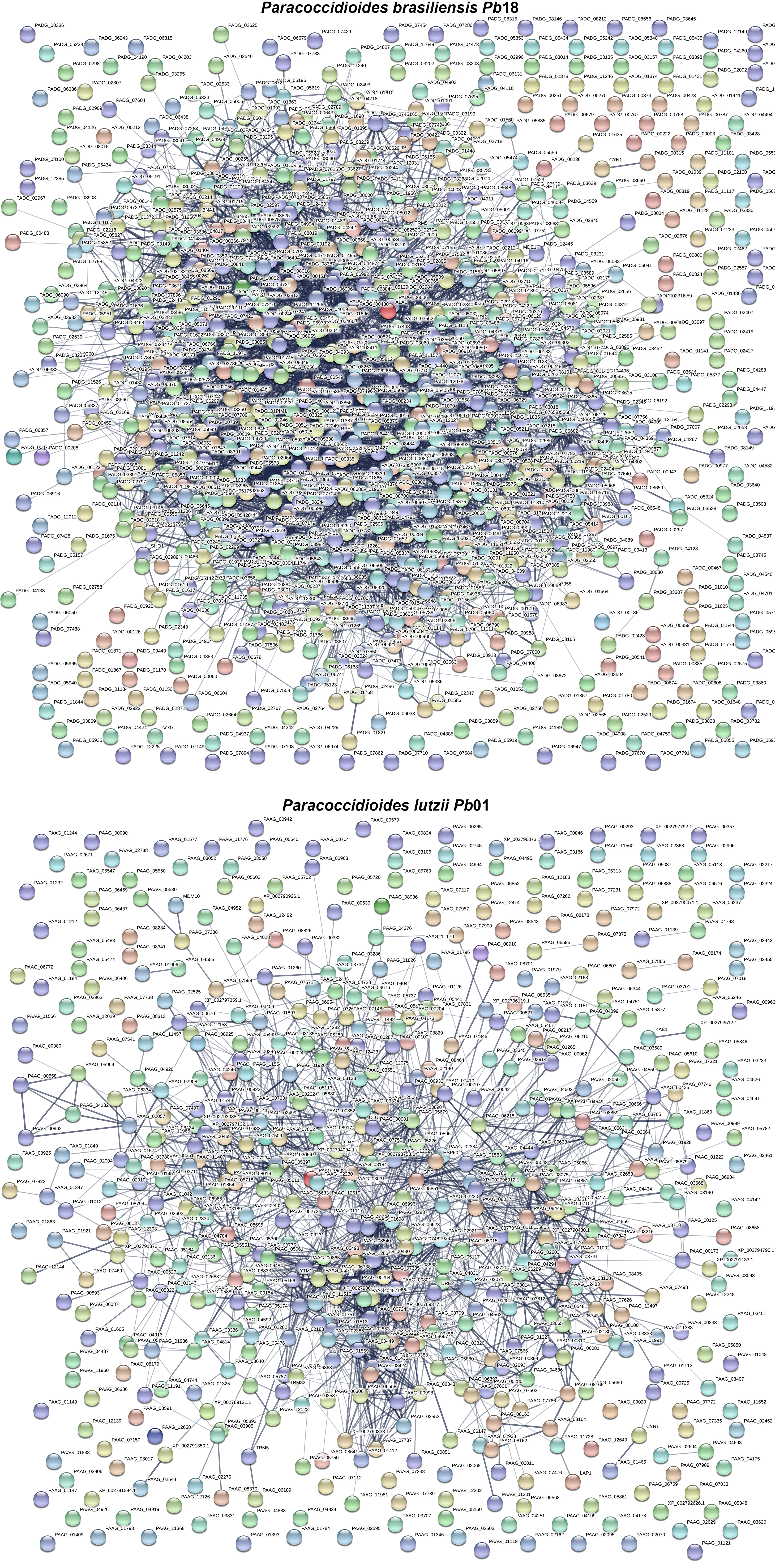

Supplement: Supplementary file 1 [file jof-06-00328-s001.zip › FigureS3.tif]

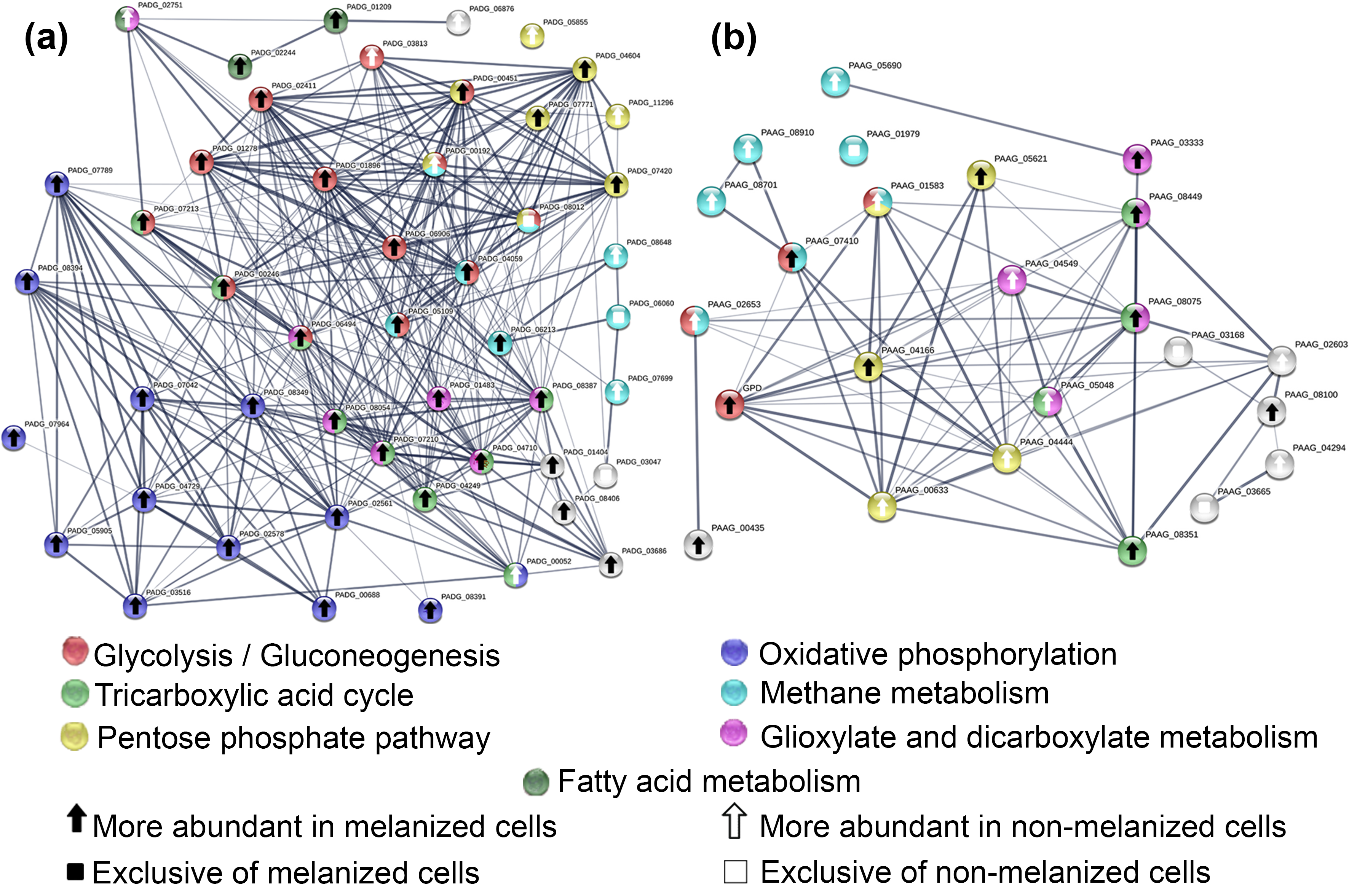

Supplement: Supplementary file 1 [file jof-06-00328-s001.zip › FigureS4.tif]

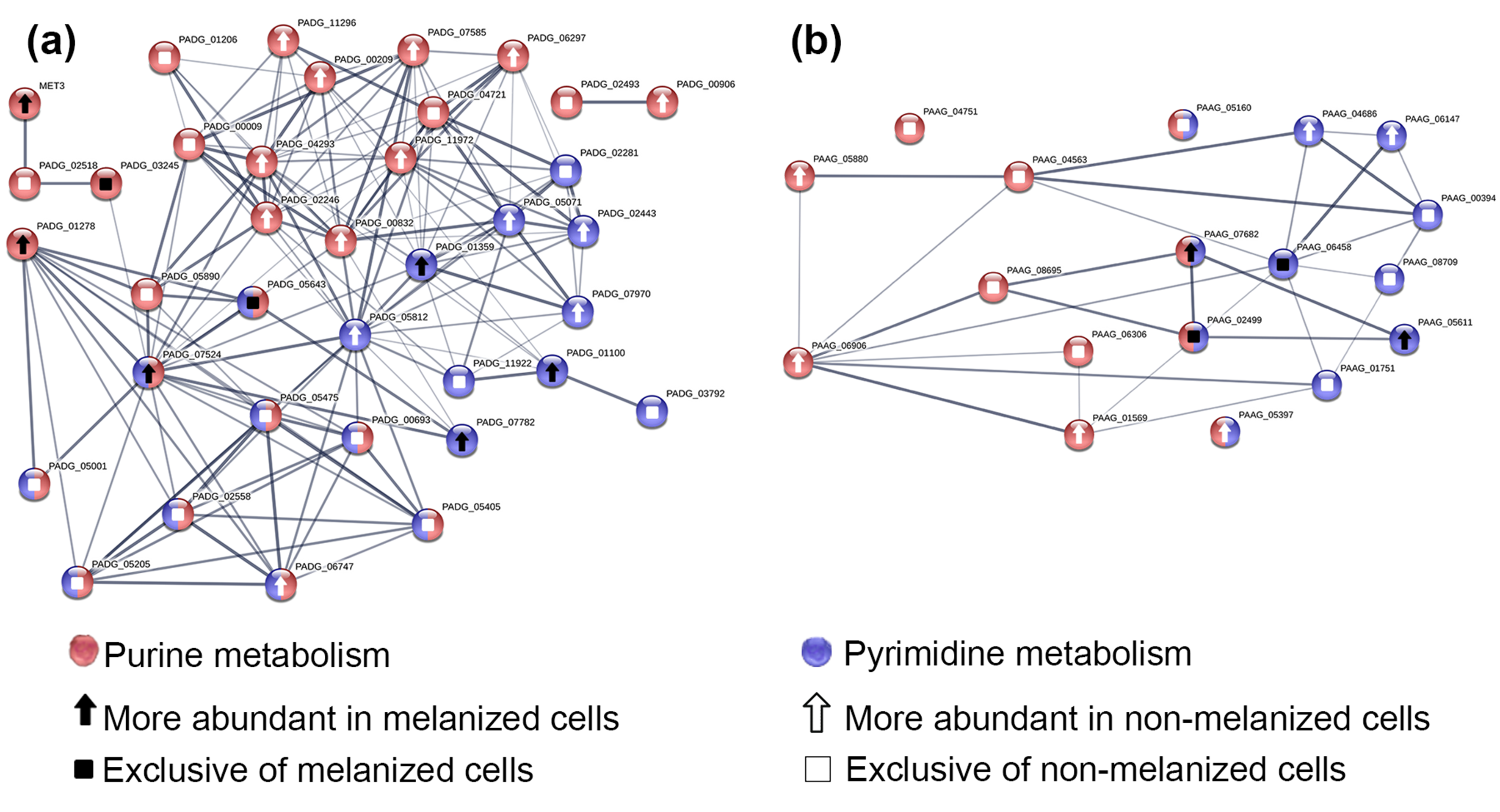

Supplement: Supplementary file 1 [file jof-06-00328-s001.zip › FigureS5.tif]

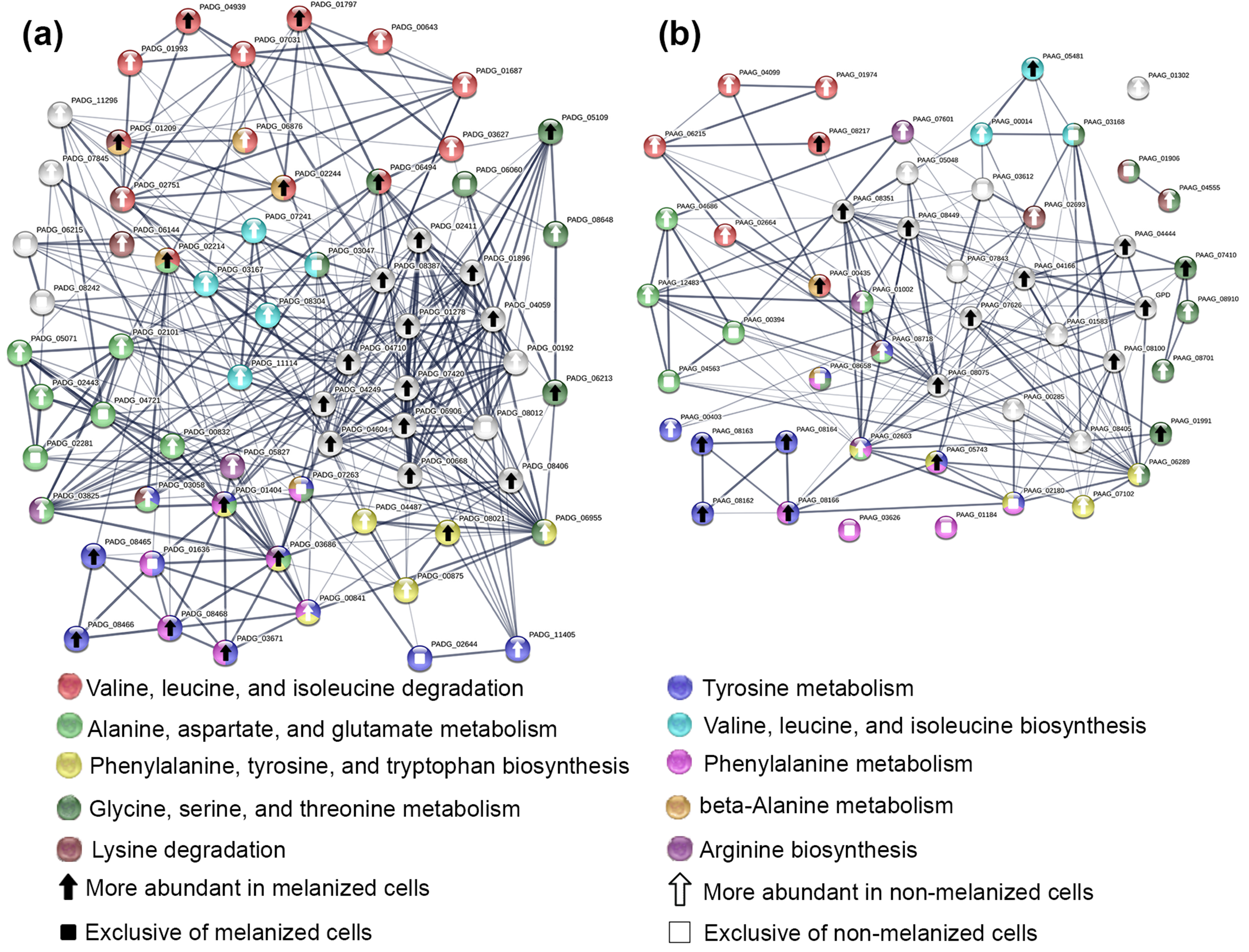

Supplement: Supplementary file 1 [file jof-06-00328-s001.zip › FigureS6.tif]

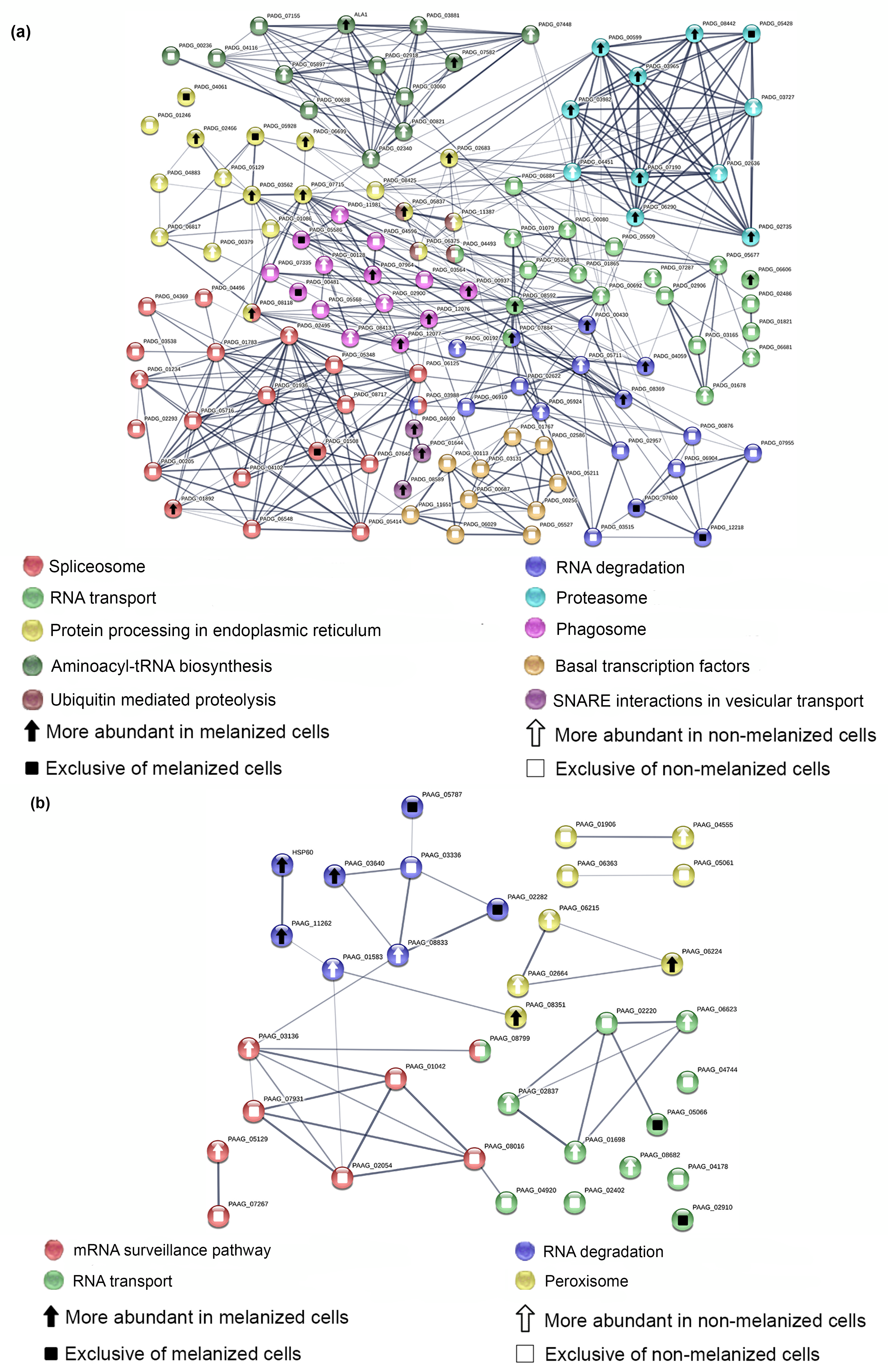

Supplement: Supplementary file 1 [file jof-06-00328-s001.zip › FigureS7.tif]

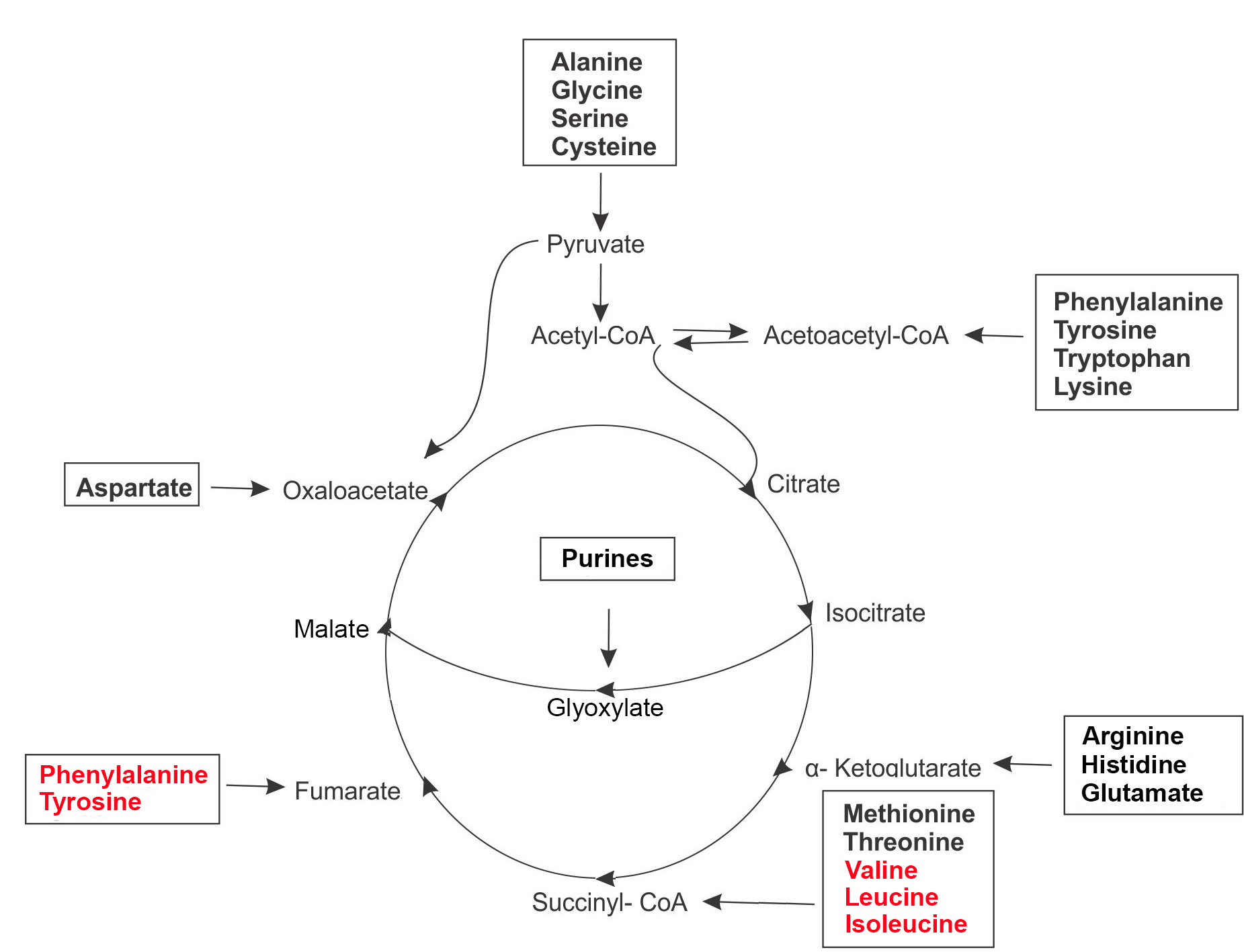

Supplement: Supplementary file 1 [file jof-06-00328-s001.zip › FigureS9.tif]
